# NUDT21 lactylation reprograms alternative polyadenylation to promote cuproptosis resistance

**DOI:** 10.1038/s41421-025-00804-1

**Published:** 2025-05-28

**Authors:** Jinlong Lin, Yixin Yin, Jinghua Cao, Yiyang Zhang, Jiewei Chen, Rixin Chen, Bingxu Zou, Cijun Huang, Yongrui Lv, Shuidan Xu, Han Yang, Peng Lin, Dan Xie

**Affiliations:** 1 State Key Laboratory of Oncology in South China, Guangdong Provincial Clinical Research Center for Cancer, Sun Yat-sen University Cancer Center, Guangzhou, Guangdong China; 2 Department of Thoracic Surgery, Sun Yat-sen University Cancer Center, Guangzhou, Guangdong China; 3 Department of Anesthesiology, Sun Yat-sen University Cancer Center, Guangzhou, Guangdong China; 4 Department of Endoscopy, Sun Yat-sen University Cancer Center, Guangzhou, Guangdong China; 5 Department of Pathology, Sun Yat-sen University Cancer Center, Guangzhou, Guangdong China; 6 Department of Thoracic Surgery, Guangdong Provincial People’s Hospital (Guangdong Academy of Medical Sciences), Southern Medical University, Guangzhou, Guangdong China

**Keywords:** Post-translational modifications, Cancer genomics, Transcription, Cell death

## Abstract

Alternative polyadenylation (APA) is critical for shaping transcriptome diversity and modulating cancer therapeutic resistance. While lactate is a well-established metabolic signal in cancer progression, its role in APA regulation remains unclear. Here, we demonstrate that l-lactate-induced lactylation of NUDT21 drives transcriptomic reprogramming through APA modulation. NUDT21 lactylation enhances its interaction with CPSF6, facilitating CFIm complex formation and inducing 3′ untranslated region (UTR) lengthening of *FDX1*. Extension of the *FDX1* 3′ UTR attenuates its protein output, thereby conferring resistance to cuproptosis in esophageal squamous cell carcinoma (ESCC). Furthermore, we identify AARS1 as the lactylation “writer” catalyzing NUDT21 K23 lactylation, and HDAC2 as its enzymatic “eraser”. Clinically, elevated levels of both LDHA and NUDT21, as well as increased K23-lactylated NUDT21, are associated with reduced FDX1 expression and worse prognosis in ESCC patients. Notably, combined targeting of the lactate-NUDT21-FDX1-cuproptosis axis with the clinical LDHA inhibitor stiripentol and the copper ionophore elesclomol synergistically suppressed tumor growth. Collectively, our work identifies lactylated NUDT21 as a critical factor linking cellular metabolism to APA and proposes a promising therapeutic strategy for ESCC treatment.

## Introduction

Alternative polyadenylation (APA) is a pervasive mechanism that generates mRNA isoforms with varying 3′ untranslated region (UTR) lengths by directing the usage of polyadenylation sites (PASs)^1,2^. It is estimated that over 70% of mammalian genes harbor multiple PASs within their 3′ UTRs^2^. The selection of a specific PAS is orchestrated by the 3′ end processing machinery, including the cleavage and polyadenylation specificity factors (CPSFs), the cleavage stimulation factors (CstFs), and the mammalian cleavage factors I and II (CFIm and CFIIm)^1^. Dysregulation of APA leads to shifts in PAS usage, which has been implicated in various human diseases, particularly cancer^3–5^. The preponderant view is that transcripts with different 3′ UTR lengths influence protein abundance by altering RNA stability, translation efficiency, or competing-endogenous RNA crosstalk^6–8^. For example, APA-mediated changes in the levels of oncogenes and tumor suppressors such as *PTEN*, *MYC*, and *CCND1* have been reported in diverse cancer types^8–10^. Despite its significance, the mechanisms underlying APA reprogramming remain poorly understood.

The Warburg effect is a cancer hallmark characterized by aerobic glycolysis^11^. Besides providing energy within the nutrient-deprived microenvironment, accelerated glycolysis produces substantial amounts of lactate, thereby promoting tumorigenesis and resistance to radiation and chemotherapy^12,13^. Recently, protein lactylation, a post-translational modification (PTM) derived from lactate, has emerged as a critical signaling in cancer biology^14^. Lactylation of proteins such as MRE11 and NBS1 facilitates DNA damage repair^13,15^, while lactylated cGAS and PD-L1 have been linked to tumor immune evasion^16,17^. However, it remains unclear whether intracellular lactate modulates APA.

Copper, an essential trace element, plays pivotal roles in redox biology, enzymatic reactions, and immune regulation^18^. However, dysregulated copper levels can be deleterious, driving a novel form of programmed cell death termed cuproptosis^19^. Cuproptosis is triggered by the direct binding of copper to mitochondrial lipoylated proteins, leading to proteotoxic stress and cell death^19^. Among the regulators of cuproptosis, ferredoxin 1 (FDX1) is essential for reducing Cu^2+^ to its more toxic form Cu^1+^ and inducing protein lipoylation^19,20^. Given that elevated serum copper levels are observed in multiple cancers, including esophageal squamous cell carcinoma (ESCC)^21^, gastric cancer^22^, and colorectal cancer^23^, elucidating the mechanisms through which tumor cells evade cuproptosis may offer a promising avenue for cancer therapy.

In this study, we uncovered a previously unrecognized mechanism linking intracellular lactate to APA regulation in ESCC. Through RNA-seq and PAS-seq analyses, we demonstrated that l-lactate induces global 3′ UTR lengthening. We identified that Nudix hydrolase 21 (NUDT21), a core component of the 3′ end processing machinery, is lactylated at K23 through the opposing activities of AARS1 and HDAC2. NUDT21-K23 lactylation enhances its interaction with CPSF6, leading to 3′ UTR lengthening of *FDX1* and cuproptosis resistance. Importantly, our results revealed that targeting the lactate-NUDT21-FDX1-cuproptosis axis with a combination of the copper ionophore elesclomol and the FDA-approved LDHA inhibitor stiripentol exerted a synergistic antitumor effect in ESCC. These findings establish a critical link between lactate-driven protein lactylation, APA regulation, and resistance to cuproptosis, offering a compelling therapeutic strategy for ESCC.

## Results

### l-lactate drives APA reprogramming in ESCC

**Fig. 1:**
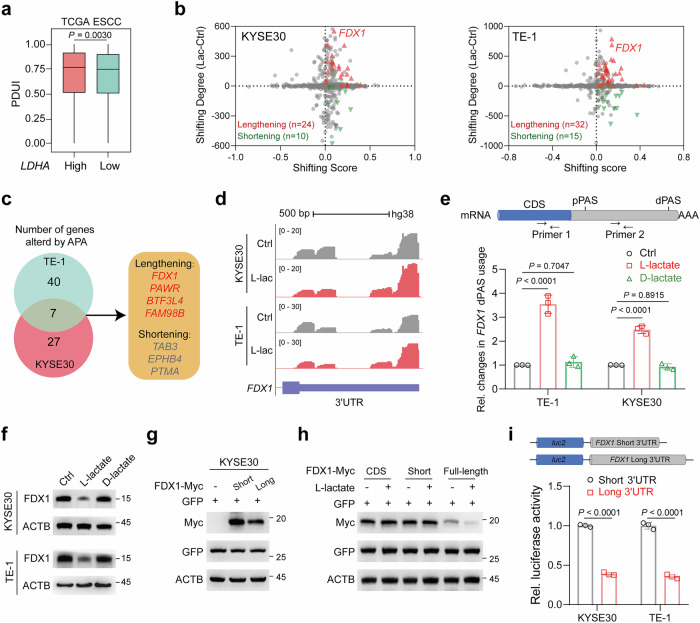
Lactylation plays a key role in APA regulation. **a** Analysis of the percentage of distal poly(A) site usage index (PDUI) in the TCGA ESCC cohort (*n* = 95). **b** Scatter plot depicting genes with lengthened (red) or shortened (green) 3′ UTRs in ESCC cells treated with l-lactate (20 mM) for 24 h. **c** Overlap of genes exhibiting 3′ UTR lengthening (*FDX1*, *PAWR*, *BTF3L4*, and *FAM98B*) in KYSE30 and TE-1 cells treated with l-lactate (20 mM) for 24 h. **d** PAS-seq density plots for *FDX1*, showing 3′ UTR lengthening in response to l-lactate. **e** Top: schematic of the pPAS and dPAS within *FDX1*, along with two primer sets designed for targeting the CDS and the distal region. Bottom: RT-qPCR analysis of *FDX1* dPAS usage in ESCC cells treated with l-lactate (20 mM) or d-lactate (20 mM) for 24 h. **f** Immunoblot analysis of FDX1 protein levels in ESCC cells following treatment with l-lactate (20 mM) or d-lactate (20 mM). **g**, **h** Immunoblot analysis of FDX1 and GFP protein levels in KYSE30 cells transfected with the indicated plasmids. **i** Top: schematic of the *FDX1* 3′ UTR luciferase reporter plasmids. Bottom: quantification of Firefly luciferase activity for *FDX1* long and short 3′ UTR constructs, normalized to *Renilla* luciferase activity. Data are presented as mean ± SD; *P* value was calculated by Mann–Whitney U test (**a**) and Student’s *t-*test (**e**, **i**).

Lactate is a well-established modulator of diverse biological processes; however, its role in APA remains largely unexplored. Intriguingly, we found that ESCC patients with elevated expression of *LDHA*, the major enzyme responsible for lactate production^13,24^, exhibited preferential usage of distal PAS (dPAS) (Fig. 1a). To investigate transcripts affected by lactate systematically, we performed PAS-seq analysis on ESCC cells treated with l-lactate, the predominant form of lactate in tumors^25,26^. PAS-seq analysis revealed that PAS sequences primarily mapped to the canonical AAUAAA motif, with clusters enriched in 3′ UTR regions (Supplementary Fig. S1a–d). Consistently, l-lactate treatment resulted in more 3′ UTR lengthening events, with 24 out of 34 (70.6%) APA events in KYSE30 cells and 32 out of 47 (68.1%) in TE-1 cells (Fig. 1b). Among the genes with 3′ UTR lengthening, *FDX1*, *PAWR*, *BTF3L4*, and *FAM98B* were identified in both cell lines (Fig. 1c, d; Supplementary Fig. S1e). Further RT-qPCR analysis using isoform-specific primers confirmed these results (Fig. 1e; Supplementary Fig. S1f–h).

Given that alternative PAS usage influences protein abundance^8,27^, we next examined the protein levels of these candidate genes. Among them, only FDX1 exhibited reduced protein levels upon l-lactate treatment (Fig. 1f; Supplementary Fig. S2a). This decline was accompanied by decreased *FDX1* mRNA expression (Supplementary Fig. S2b), and neither autophagy nor proteasome inhibition restored FDX1 protein levels (Supplementary Fig. S2c–e), indicating that l-lactate destabilizes *FDX1* mRNA. To determine whether this reduction was driven by dPAS selection, we constructed two *FDX1* vectors encoding either the short or long 3′ UTR. Notably, the vector containing the long *FDX1* 3′ UTR incorporated a mutated proximal PAS (pPAS) to enforce exclusive dPAS usage. As shown in Fig. 1g and Supplementary Fig. S2f, dPAS usage significantly attenuated FDX1 protein output. Moreover, only isoforms harboring the full-length 3′ UTR responded to l-lactate, whereas those containing only the coding sequence (CDS) region or a short 3′ UTR remained unaffected (Fig. 1h; Supplementary Fig. S2g). Consistently, dual-luciferase reporter assays indicated that the long 3′ UTR of *FDX1* reduced luciferase activity (Fig. 1i), further confirming that l-lactate-mediated APA reprogramming decreases FDX1 protein levels in ESCC.

Lactate exists as two enantiomers, l-lactate and d-lactate, with mammalian LDHA demonstrating stereoselectivity for l-lactate^28^. To assess whether d-lactate exerts similar effects on *FDX1*, we treated ESCC cells with d-lactate. Unlike l-lactate, d-lactate failed to regulate the 3′ UTR length of *FDX1* or its protein abundance (Fig. 1e, f), suggesting that the regulation of *FDX1* by lactate is specific to l-lactate.

### l-lactate promotes NUDT21 lactylation

**Fig. 2:**
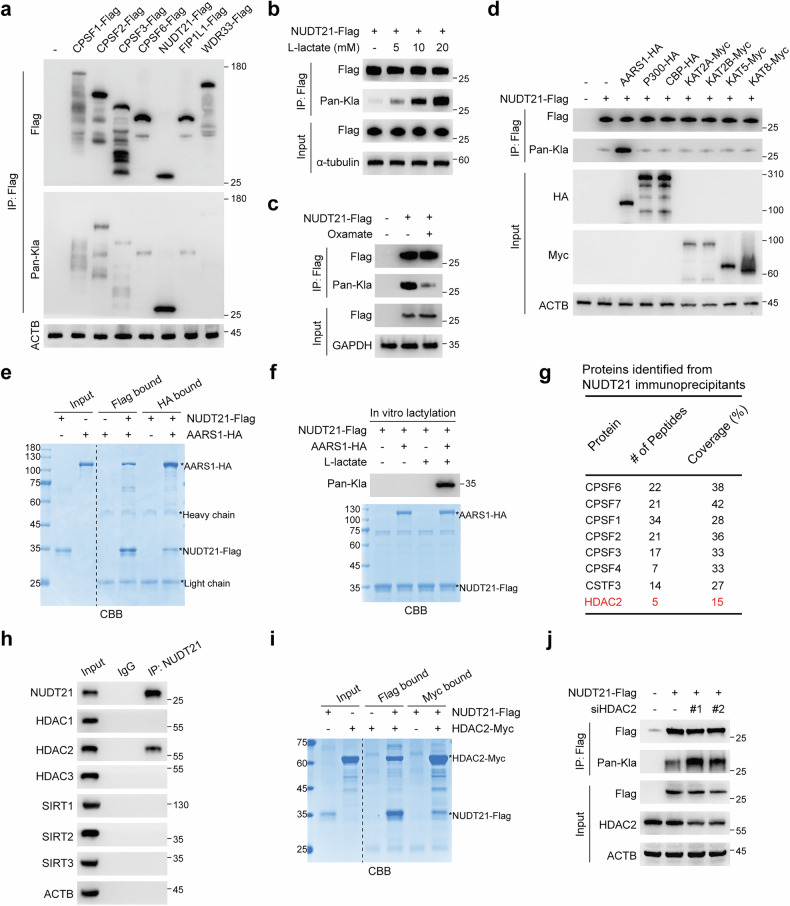
AARS1 and HDAC2 mediate NUDT21 lactylation. **a** Immunoblot analysis of lactylation levels in core proteins involved in 3′ UTR processing. **b** Immunoblot analysis of NUDT21 lactylation levels in KYSE30 cells treated with increasing concentrations of l-lactate. **c** Immunoblot analysis of NUDT21 lactylation levels in KYSE30 cells treated with oxamate (20 mM). **d** Screening for the enzyme(s) responsible for NUDT21 lactylation. **e** In vitro reciprocal pull-down assays confirming the direct interaction between NUDT21 and AARS1, visualized by Coomassie Brilliant Blue (CBB) staining. **f** In vitro lactylation assay detecting the lactylation of NUDT21 catalyzed by AARS1. CBB staining shows purified NUDT21-Flag and AARS1-HA proteins used in the assay. **g** Table summarizing key proteins identified by mass spectrometry analysis, with the protein of interest highlighted in red font. **h**, **i** The interaction between NUDT21 and HDAC2 was detected by Co-IP in 293 T cells (**h**) and in vitro reciprocal pull-down assays (**i**). **j** Immunoblot analysis of NUDT21 lactylation levels in KYSE30 cells transfected with siRNAs specific for HDAC2.

Protein lactylation, a modification driven by l-lactate, is increasingly recognized as a pivotal post-translational signal regulating diverse biological processes^29^. To investigate whether l-lactate mediates APA through lactylation, we integrated lactylomic datasets from multiple studies and identified several core components of the 3′ end processing machinery as potential lactylation targets^29–31^ (Supplementary Fig. S3a). Among these, NUDT21 (also known as CFIm25) exhibited significant lactylation (Fig. 2a). Furthermore, l-lactate treatment induced a concentration-dependent increase of lactylated NUDT21 (Fig. 2b), whereas inhibition of LDHA by oxamate markedly attenuated NUDT21 lactylation (Fig. 2c).

Next, we sought to identify the potential “writer” responsible for NUDT21 lactylation. Among several known lactyltransferases, AARS1 was uniquely capable of enhancing NUDT21 lactylation (Fig. 2d). Direct interaction between AARS1 and NUDT21 was validated by co-immunoprecipitation (Co-IP) and pull-down assays (Fig. 2e; Supplementary Fig. S3b). In vitro lactylation assays further confirmed AARS1 as the enzyme responsible for catalyzing NUDT21 lactylation (Fig. 2f). To delineate the regulatory nexus of NUDT21 lactylation, we performed LC-MS/MS analysis following NUDT21 immunoprecipitation to identify potential delactylases. In addition to other subunits of the 3′ end processing machinery, we identified HDAC2, a member of the class I histone deacetylases (HDAC1–3) implicated in lysine delactylation^32^, as a novel binding partner of NUDT21 (Fig. 2g). The interaction between NUDT21 and HDAC2, but not other known delactylases, was substantiated by Co-IP and reciprocal pull-down assays (Fig. 2h, i; Supplementary Fig. S3c, d). Additionally, knockdown of HDAC2 significantly increased NUDT21 lactylation levels (Fig. 2j). Collectively, these findings establish that l-lactate drives NUDT21 lactylation through the coordinated actions of AARS1 and HDAC2.

### NUDT21 K23 lactylation promotes dPAS usage of *FDX1*

**Fig. 3:**
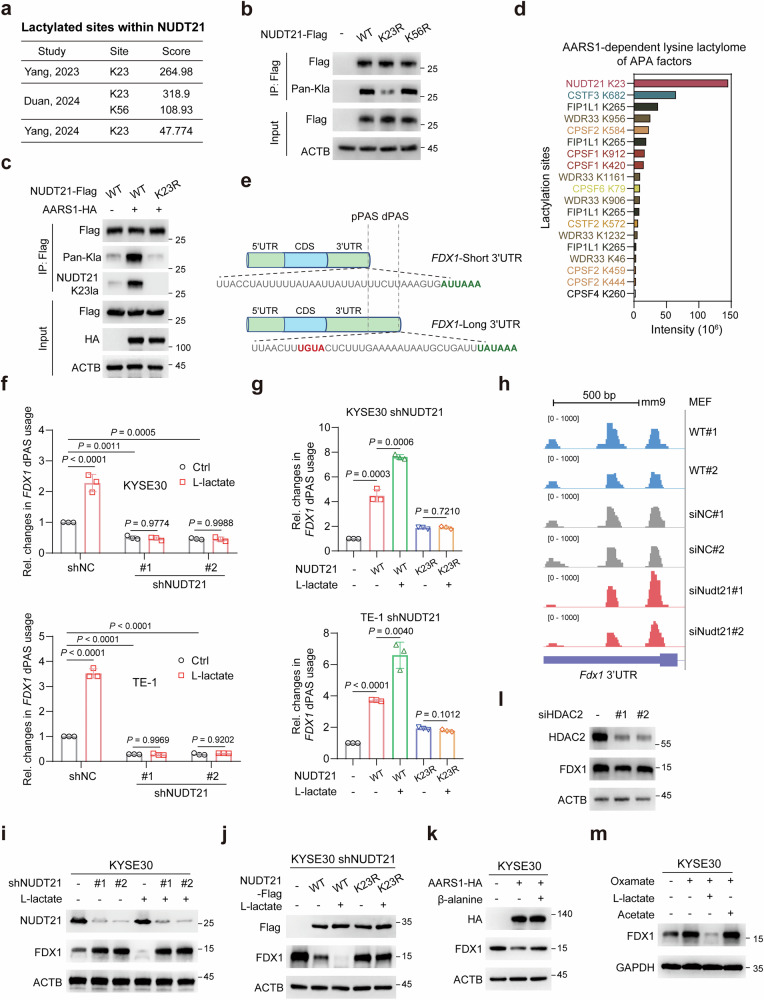
NUDT21 K23 lactylation promotes 3′ UTR lengthening of *FDX1.* **a** Table summarizing the lactylation sites of NUDT21 identified through three independent studies. **b** Immunoblot analysis of NUDT21 lactylation levels in KYSE30 cells transfected with the indicated NUDT21 mutant plasmids. **c** Immunoblot analysis of AARS1-induced NUDT21 lactylation in 293T cells transfected with either WT NUDT21 or K23R mutant. **d** In vitro AARS1-dependent lysine lactylation of APA factors identified from 293T cell lysates, incubated with AARS1, l-lactate, and ATP. **e** Schematic illustrating the PAS (green font) and the NUDT21-binding motif (red font) within *FDX1*. **f** RT-qPCR analysis of *FDX1* dPAS usage in NUDT21-knockdown ESCC cells treated with l-lactate (20 mM) for 24 h. **g** RT-qPCR analysis of *FDX1* dPAS usage in NUDT21-knockdown ESCC cells transfected with WT NUDT21 or K23R mutant, with or without l-lactate (20 mM) stimulation for 24 h. **h** PAS-seq density plots for *Fdx1*, showing 3′ UTR shortening in response to NUDT21 knockdown in the GSE99922 dataset. **i** Immunoblot analysis of FDX1 protein levels following NUDT21 knockdown in KYSE30 cells, with or without l-lactate (20 mM) stimulation for 24 h. **j** Immunoblot analysis of FDX1 protein levels in NUDT21-knockdown KYSE30 cells transfected with WT NUDT21 or K23R mutant, with or without l-lactate (20 mM) stimulation for 24 h. **k** Immunoblot analysis of FDX1 protein levels in KYSE30 cells transfected with AARS1-HA, with or without β-alanine (100 mM) stimulation for 24 h. **l** Immunoblot analysis of FDX1 protein levels in KYSE30 cells transfected with siRNAs specific for HDAC2. **m** Immunoblot analysis of FDX1 protein levels in KYSE30 cells treated with oxamate (20 mM), l-lactate (20 mM), or acetate (10 mM) for 24 h. Data are presented as mean ± SD; *P* value was calculated by Student’s *t*-test (**f**, **g**).

Previous studies have indicated that several components of the 3′ end processing machinery can undergo lactylation, with residues K23 and K56 identified as potential lactylation sites on NUDT21^29–31^ (Fig. 3a; Supplementary Fig. S4a–f). Through site-directed mutants, we found that K23 serves as the primary lactylation site (Fig. 3b). Consistently, AARS1 failed to enhance lactylation of the NUDT21 K23R mutant (Fig. 3c). Using a custom antibody against K23-lactylated NUDT21 further confirmed K23 lactylation following l-lactate treatment or AARS1 overexpression (Fig. 3c; Supplementary Fig. S4g, h). Supporting this conclusion, recent proteomic profiling has identified NUDT21 as a direct target of AARS1-mediated lactylation, with K23 exhibiting the highest lactylation levels among 3′ end processing subunits^33^ (Fig. 3d; Supplementary Fig. S4i). These results establish K23 as the principal site of AARS1-dependent NUDT21 lactylation.

NUDT21 recognizes the UGUA motif to facilitate downstream PAS selection and 3′ end formation^34^. Sequence analysis of *FDX1* revealed a UGUA motif upstream of the dPAS but not the pPAS, suggesting that NUDT21 preferentially drives dPAS usage in *FDX1* (Fig. 3e). Indeed, NUDT21 depletion led to 3′ UTR shortening of *FDX1* (Fig. 3f), while restoration of NUDT21 shifted PAS usage toward the distal site (Fig. 3g). Importantly, l-lactate failed to induce dPAS usage in the absence of NUDT21 (Fig. 3f), indicating a NUDT21-dependent mechanism. Furthermore, l-lactate enhanced the ability of wild-type (WT) NUDT21 to promote *FDX1* dPAS usage, while this effect was abolished in cells expressing the NUDT21 K23R mutant (Fig. 3g). Consistently, PAS-seq conducted in mouse embryonic fibroblasts (MEFs) revealed significant 3′ UTR shortening of *Fdx1* upon NUDT21 ablation^35^ (Fig. 3h; Supplementary Fig. S4j). These findings collectively demonstrate that l-lactate increases the dPAS isoform of *FDX1* via NUDT21 K23 lactylation.

Given the functional significance of APA in regulating FDX1 protein output, we next examined the impact of NUDT21 on FDX1 protein levels. As expected, NUDT21 depletion elevated FDX1 protein levels, even in the presence of l-lactate (Fig. 3i; Supplementary Fig. S5a, b). Conversely, in NUDT21 WT cells, l-lactate further reduced FDX1 protein levels, an effect that was abolished in NUDT21 K23R cells (Fig. 3j; Supplementary Fig. S5c, d), underscoring the critical role of K23 lactylation in this regulation. Similarly, AARS1 mitigated FDX1 protein levels, while inhibiting AARS1 lactyltransferase activity with β-alanine, which competes with lactate for binding to AARS1^33^, reversed this effect (Fig. 3k). Moreover, HDAC2 depletion led to a reduction in FDX1 protein levels (Fig. 3l), whereas AARS1 knockdown or HDAC2 overexpression yielded opposing effects (Supplementary Fig. S5e, f). Notably, these regulatory effects were lost upon NUDT21 depletion or K23R mutation (Supplementary Fig. S5g, h). Taken together, these results suggest that NUDT21 K23 lactylation enhances its regulation of FDX1.

It has been reported that NUDT21 K23 is also subjected to acetylation, which impairs its interaction with poly(A) polymerase (PAP)^36^. To investigate whether NUDT21 acetylation directly influences *FDX1* APA, we pre-treated cells with oxamate to suppress endogenous lactate production, leading to increased FDX1 protein levels. As shown in Fig. 3m and Supplementary Fig. S5i, l-lactate diminished FDX1 levels, while acetate, a precursor for acetylation^37^, had minimal impact. These results suggest that NUDT21 lactylation, rather than acetylation, serves as the primary regulatory mechanism for FDX1.

### Lactylation of NUDT21 facilitates CFIm complex formation

**Fig. 4:**
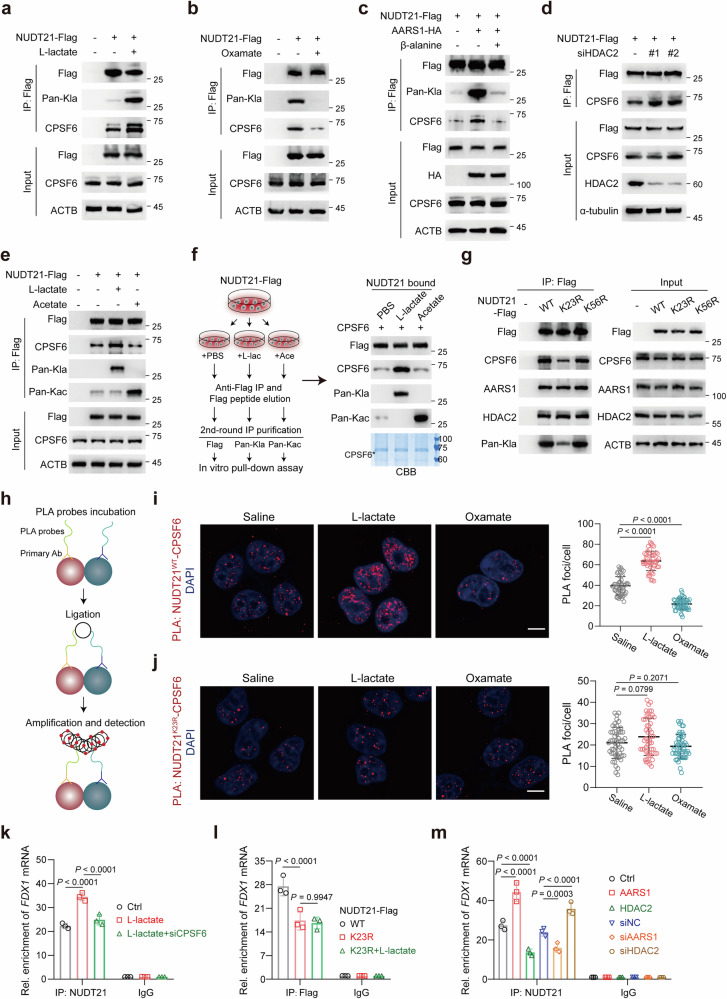
Lactylation of NUDT21 facilitates NUDT21–CPSF6 complex formation. **a**, **b** Immunoblot analysis of the interaction between NUDT21 and CPSF6 in KYSE30 cells treated with 20 mM l-lactate (**a**) or 20 mM oxamate (**b**). **c** Immunoblot analysis of the interaction between NUDT21 and CPSF6 in KYSE30 cells transfected with AARS1-HA, with or without β-alanine (100 mM) stimulation for 24 h. **d** Immunoblot analysis of the interaction between NUDT21 and CPSF6 in KYSE30 cells transfected with siRNA specific for HDAC2. **e** Immunoblot analysis of the interaction between NUDT21 and CPSF6 in KYSE30 cells treated with acetate (10 mM) or l-lactate (20 mM). Cells were pre-treated with oxamate. **f** In vitro pull-down assays detecting the interaction between CPSF6 and NUDT21. Left: schematic of purification. Cells were pre-treated with oxamate before l-lactate or acetate treatment. **g** Immunoblot analysis of the interaction between NUDT21 and its binding partners in KYSE30 cells transfected with the indicated plasmids. **h** Experimental schematic of the PLA assay. **i**, **j** Representative images of PLA foci showing the interaction between NUDT21 and CPSF6 in KYSE30 cells treated with saline, l-lactate (20 mM), or oxamate (20 mM). Scale bars, 5 μm. The right panel is quantification, *n* = 50 cells. **k**–**m** RIP assays assessing the association between NUDT21 and *FDX1* RNA in KYSE30 cells under the indicated conditions. Data are presented as mean ± SD; *P* value was calculated by Student’s *t*-test (**i**–**m**).

The interaction between NUDT21 and CPSF6, which forms the CFIm complex, represents a critical early event in APA, facilitating the recruitment and assembly of the 3′ end processing machinery^2,38,39^. Therefore, we investigated whether NUDT21 lactylation modulates CFIm complex formation. Co-IP assays revealed that l-lactate markedly enhanced the interaction between NUDT21 and CPSF6 (Fig. 4a), whereas oxamate impaired this interaction (Fig. 4b). Similarly, AARS1 overexpression or HDAC2 knockdown significantly promoted the NUDT21–CPSF6 interaction, while β-alanine treatment, AARS1 depletion, or HDAC2 overexpression had the opposite effect (Fig. 4c, d; Supplementary Fig. S6a). By contrast, acetate treatment, which robustly increased NUDT21 acetylation, had no impact on CFIm complex formation (Fig. 4e). In vitro pull-down assays further confirmed that CPSF6 preferentially interacted with lactylated NUDT21, rather than its acetylated or non-lactylated forms (Fig. 4f). Consistently, the NUDT21 K23R mutant, but not the K56R mutant, exhibited reduced binding affinity for CPSF6 (Fig. 4g), and this effect was not influenced by AARS1 or HDAC2 (Supplementary Fig. S6b). These findings were corroborated by proximity ligation assay (PLA), which visualized in situ NUDT21–CPSF6 interaction as nuclear foci (Fig. 4h). As shown in Fig. 4i, PLA foci increased upon l-lactate treatment but were diminished in response to oxamate. However, no significant changes in PLA foci were observed in NUDT21 K23R cells, regardless of l-lactate or oxamate treatment (Fig. 4j), underscoring the role of K23 lactylation in facilitating the NUDT21–CPSF6 interaction.

The interaction with CPSF6 enhances the RNA-binding ability of NUDT21^38^. We next examined whether NUDT21 lactylation regulates its *FDX1*-binding capacity using RNA immunoprecipitation (RIP)-qPCR. Our results showed that NUDT21 could bind to *FDX1* RNA, and this interaction was enhanced following l-lactate treatment (Fig. 4k), an effect that was abolished in NUDT21 K23R mutant (Fig. 4l). Consistently, AARS1 overexpression or HDAC2 knockdown promoted the NUDT21–*FDX1* interaction, whereas AARS1 depletion or HDAC2 overexpression exerted opposing effect (Fig. 4m). Notably, CPSF6 knockdown abrogated the l-lactate-driven increase in NUDT21–*FDX1* binding (Fig. 4k; Supplementary Fig. S6c), suggesting that lactylation enhances the enrichment of NUDT21 on *FDX1* in a CPSF6-dependent manner.

### Lactylation of NUDT21 confers resistance to cuproptosis by targeting *FDX1*

**Fig. 5:**
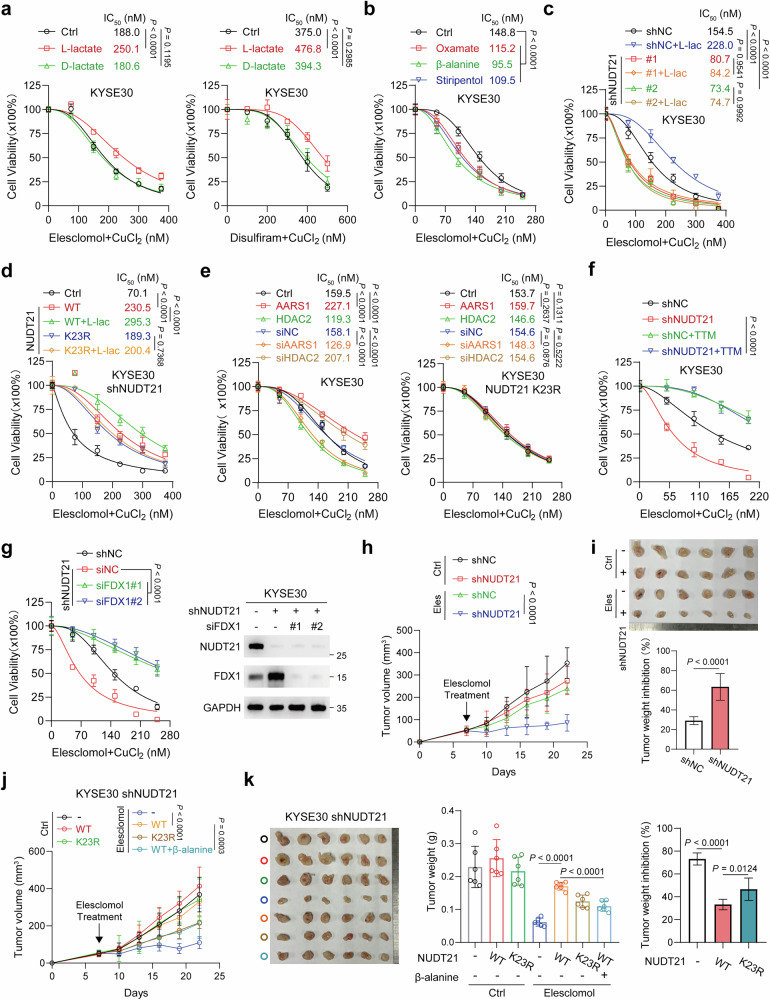
Lactylation of NUDT21 enhances resistance to cuproptosis by targeting *FDX1.* **a**, **b** The cell viability of KYESE30 cells treated with l-lactate, d-lactate (**a**), oxamate, β-alanine, or stiripentol (**b**) in response to elesclomol-Cu^2+^ or disulfiram-Cu^2+^, as measured by CCK8. **c** The cell viability of NUDT21-knockdown KYSE30 cells following treatment with elesclomol-Cu^2+^, with or without l-lactate (20 mM), as measured by CCK8. **d** The cell viability of NUDT21 WT- or K23R-overexpressing KYSE30 cells was measured by CCK8 after treatment with elesclomol-Cu^2+^, with or without l-lactate (20 mM). **e** The cell viability of KYESE30 cells transfected with the indicated plasmids or siRNAs in response to elesclomol-Cu^2+^, as measured by CCK8. **f**, **g** TTM (1 μM) treatment (**f**) or FDX1 depletion (**g**) reversed the growth inhibition induced by NUDT21 knockdown in KYSE30 cells treated with elesclomol-Cu^2+^. **h**, **i** Subcutaneous tumors of nude mice with control or NUDT21-knockdown KYSE30 cells (*n* = 6). Mice were treated with or without elesclomol. Tumor volume (**h**) and weight (**i**) were measured. **j**, **k** Subcutaneous tumors of nude mice with NUDT21 WT- or K23R-overexpressing KYSE30 cells (*n* = 6). Mice were treated with or without elesclomol. Tumor volume (**j**) and weight (**k**) were measured. Data are presented as mean ± SD; *P* value was calculated by two-way ANOVA (**a**–**h**, **j**) and Student’s *t*-test (**i**, **k**).

Given the central role of FDX1 in modulating cuproptosis^18^, we investigated whether lactylated NUDT21 affects cellular sensitivity to copper-induced stress in ESCC. Treatment with copper ionophores elesclomol or disulfiram combined with Cu²⁺ (1:1 ratio) induced significant cell death, while l-lactate conferred resistance (Fig. 5a; Supplementary Fig. S7a–c). By contrast, d-lactate failed to elicit a protective effect (Fig. 5a; Supplementary Fig. S7c). Moreover, ESCC cells exhibited lower IC_50_ values for elesclomol-Cu^2+^ upon oxamate or β-alanine treatment (Fig. 5b; Supplementary Fig. S7d). Similarly, stiripentol, an LDHA inhibitor used clinically for Dravet syndrome^15,40^, sensitized cells to copper-induced cytotoxicity (Fig. 5b; Supplementary Fig. S7d), whereas acetate exerted no effect (Supplementary Fig. S7e). NUDT21 depletion markedly reduced IC_50_ values for elesclomol/disulfiram-Cu²⁺, an effect that l-lactate treatment failed to rescue (Fig. 5c; Supplementary Fig. S8a, b). Furthermore, l-lactate enhanced resistance in NUDT21 WT cells but not in NUDT21 K23R cells (Fig. 5d; Supplementary Fig. S8c, d). Consistently, AARS1 overexpression or HDAC2 depletion restored cell viability under copper-induced stress, whereas AARS1 knockdown or HDAC2 overexpression exacerbated cuproptosis (Fig. 5e; Supplementary Fig. S8e–g). Notably, these effects were observed in NUDT21 WT cells, with no impact on K23R cells (Fig. 5e; Supplementary Fig. S8e–g). Treatment with tetrathiomolybdate (TTM), a cuproptosis inhibitor, effectively suppressed copper-induced cell death in both control and NUDT21-knockdown cells (Fig. 5f; Supplementary Fig. S8h), confirming NUDT21’s role in cuproptosis regulation. Additionally, FDX1 depletion reversed cuproptosis induced by NUDT21 knockdown (Fig. 5g; Supplementary Fig. S9a–c), indicating that lactylated NUDT21 inhibits cuproptosis through FDX1 suppression.

In subcutaneous xenograft models, elesclomol significantly inhibited tumor growth formed by NUDT21-knockdown KYSE30 cells (Fig. 5h, i; Supplementary Fig. S9d). Notably, the enhanced sensitivity of NUDT21-depleted tumors to elesclomol was abrogated following FDX1 depletion (Supplementary Fig. S9e, f). Restoration of WT NUDT21 markedly enhanced resistance to elesclomol, while co-treatment with β-alanine reduced tumor volumes and weights, comparable to tumors harboring the NUDT21 K23R mutant (Fig. 5j, k). Importantly, copper levels remained unchanged in both NUDT21-knockdown and K23R cells, underscoring the specific role of NUDT21 in modulating cuproptosis sensitivity (Supplementary Fig. S9g). These findings establish NUDT21 lactylation as a critical regulator of cuproptosis resistance in ESCC.

### Lactate-NUDT21-FDX1-cuproptosis axis is a promising target for ESCC treatment

**Fig. 6:**
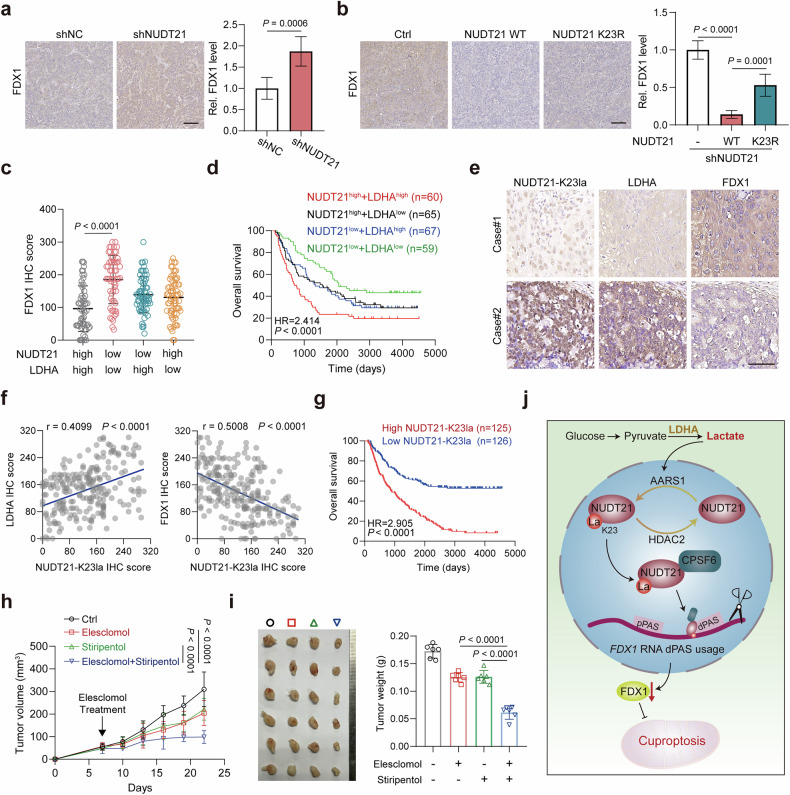
The lactate-NUDT21-FDX1-cuproptosis axis represents a promising therapeutic target for ESCC. **a**, **b** Subcutaneous tumors were subjected to FDX1 staining. Scale bars, 100 μm. Quantitative results of FDX1 staining were presented (*n* = 6). **c** Quantitative results of FDX1 staining in ESCC samples stratified by NUDT21 and LDHA levels (*n* = 60, 65, 67, and 59, respectively). **d** High levels of NUDT21 and LDHA are correlated with the lowest overall survival rate in the ESCC cohort. **e** Representative IHC images of K23-lactylated NUDT21, LDHA, and FDX1 staining in serial ESCC sections. Scale bars, 100 μm. **f** Scatter plots illustrating a positive correlation between LDHA and K23-lactylated NUDT21 levels (left) and a negative correlation between FDX1 and K23-lactylated NUDT21 levels (right) in the ESCC cohort (*n* = 251). **g** Elevated levels of K23-lactylated NUDT21 are associated with reduced overall survival rate in the ESCC cohort (*n* = 251). **h**, **i** Subcutaneous tumors of nude mice with KYSE30 cells (*n* = 6). Mice were treated with saline, elesclomol, stiripentol, or elesclomol + stiripentol. Tumor volume (**h**) and weight (**i**) were measured. **j** Schematic model illustrating the role of the lactate-NUDT21-FDX1-cuproprosis axis in ESCC. Data are presented as mean ± SD; *P* value was calculated by Student’s *t*-test (**a**–**c**, **i**), log-rank test (**d**, **g**), Pearson correlation analysis (**f**), and two-way ANOVA (**h**).

To explore the clinical relevance of lactylated NUDT21 and FDX1, we analyzed the UCSC xena dataset. Transcriptome profiling revealed negative correlations between *FDX1* expression and key molecules associated with NUDT21 lactylation, including *NUDT21*, *LDHA*, and *AARS1* (Supplementary Fig. S10a). By contrast, a positive correlation between *FDX1* and *HDAC2* suggested their functional convergence within the lactate-NUDT21-FDX1-cuproptosis axis (Supplementary Fig. S10b). Consistently, immunohistochemistry (IHC) analysis of tumor sections showed elevated FDX1 levels in NUDT21-knockdown xenografts (Fig. 6a). Restoration of WT NUDT21 significantly reduced FDX1 staining compared to control and NUDT21 K23R groups (Fig. 6b), underscoring the regulatory impact of NUDT21 lactylation on FDX1 expression. Clinically, patients with high *LDHA* and *NUDT21* expressions exhibited substantially lower *FDX1* levels and poorer overall survival compared to other groups (Supplementary Fig. S10c, d). These observations were validated by IHC in a cohort of 251 clinical ESCC cases, where elevated NUDT21 and LDHA levels correlated with reduced FDX1 expression and unfavorable prognosis (Fig. 6c, d; Supplementary Fig. S10e). Notably, NUDT21 K23 lactylation positively correlated with LDHA and inversely with FDX1, and patients with high levels of K23-lactylated NUDT21 exhibited significantly worse survival (Fig. 6e–g), further reinforcing the clinical significance of this regulatory axis.

Comprehensive analysis of TCGA datasets also revealed widespread dysregulation of copper homeostasis pathways in ESCC, evidenced by aberrant expression of genes involved in copper uptake, transport, and utilization^18^ (Supplementary Fig. S10f). Additionally, the majority of metallothionein proteins, critical for copper storage and sequestration^18^, were significantly downregulated in ESCC tissues (Supplementary Fig. S10g), indicating a compromised copper buffering capacity. Given the upregulated expressions of *NUDT21* and *LDHA* in ESCC and their association with cuproptosis resistance (Supplementary Fig. S10h), targeting lactylated NUDT21 emerges as a prospective strategy. To explore this potential, we evaluated the combined treatment of elesclomol and stiripentol, which demonstrated pronounced synergistic antitumor effects (Fig. 6h, i). Furthermore, the combination treatment was well-tolerated throughout the therapeutic course (Supplementary Fig. S10i). Collectively, these results reveal an intrinsic vulnerability to cuproptosis in ESCC and identify the lactate-NUDT21-FDX1 axis as a promising target for copper-based cancer therapeutics.

## Discussion

Enhanced glycolysis and consequent lactate accumulation are hallmark features of various cancers^41^. While lactate’s oncogenic properties have been recognized for decades, its molecular mechanisms remain incompletely defined. Lactylation, a recently characterized PTM, was initially implicated in epigenetic regulation via histone modification^14^. Expanding this concept, integrative proteomic analyses have revealed lactylation of non-histone proteins with diverse regulatory functions. For instance, lactylation enhances MRE11’s DNA-binding affinity^13^, while repressing the DNA-binding and liquid–liquid phase separation of p53 and cGAS^16,33^. Moreover, lactate strengthens the YAP–TEAD1 interaction and promotes MRN complex formation through NBS1 lactylation^15,42^. Herein, we identified NUDT21 as a novel lactylation substrate at K23, orchestrated by the opposing activities of AARS1 and HDAC2. Site-specific lactylation of NUDT21 enhances its interaction with CPSF6, thereby strengthening its RNA-binding affinity for *FDX1*. Notably, the NUDT21 K23R mutant retains partial regulatory capacity over *FDX1*, albeit to a lesser extent than WT NUDT21, likely reflecting the basal activity of non-lactylated NUDT21 or the involvement of alternative regulatory mechanisms. Interestingly, while previous reports identified K23 acetylation as crucial for NUDT21’s interaction with PAP^36^, we found that acetylation had a minimal direct impact on CPSF6 binding. However, competitive modifications at K23 may indirectly disrupt the NUDT21–CPSF6 interaction.

d-lactate, a stereoisomer of l-lactate generated through the glyoxalase pathway and LDHD activity, has also been implicated in lactylation processes, termed D-lactylation or lactoylation^25,43,44^. Notably, mitochondrial d-lactate has been linked to ferroptosis resistance in ESCC^45^. In our study, we observed that l-lactate, but not d-lactate, promoted dPAS usage of *FDX1* through NUDT21 lactylation, conferring resistance to cuproptosis in ESCC. These findings align with previous reports suggesting distinct functional roles for L- and d-lactate in cellular signaling^26,28^. However, whether D-lactylation occurs on NUDT21 and its potential regulatory effects remain to be elucidated.

Despite belonging to the Nudix hydrolase family, NUDT21 lacks enzymatic activity due to its α-helix loop motif, which blocks the catalytic site^46^. Instead, its primary function lies in the 3′ end processing of pre-mRNAs, promoting dPAS selection^34,35,47^. In line with this, we demonstrated that l-lactate treatment in ESCC cells shifts PAS usage toward more distal sites, particularly within *FDX1*, and depletion of NUDT21 abolishes the effect of l-lactate on *FDX1* 3′ UTR lengthening. Notably, NUDT21 exhibits the highest lactylation levels among the subunits of the 3′ end processing machinery, suggesting its central role in l-lactate-driven global 3′ UTR remodeling and other potential regulatory effects. Moreover, as other machinery subunits also undergo lactylation, the broader impact of lactate on APA reprogramming and ESCC progression cannot be ruled out.

The role of NUDT21 in cancer remains controversial. While its tumor-suppressive effects have been documented in glioblastoma, cervical, bladder, and hepatocellular carcinomas^35,48–50^, evidence from pancreatic cancer, head and neck squamous cell carcinoma, and gastric cancer supports an oncogenic role^51–53^. However, its biological functions in ESCC remain unexplored. Here, we reveal an oncogenic function of NUDT21 in ESCC mediated through l-lactate-driven PTM. Specifically, lactylation of NUDT21 increases the dPAS isoform of *FDX1*, reducing its protein output and thereby promoting resistance to copper-induced cell death. Clinical analyses further confirmed that patients with elevated K23-lactylated NUDT21 levels have worse prognoses, highlighting the synergistic effect of NUDT21 and lactate in driving ESCC progression.

Cuproptosis, a distinct form of cell death induced by copper overload, differs from other regulated cell death pathways such as apoptosis, pyroptosis, ferroptosis, and necroptosis^19^. While most studies have focused on inducing cuproptosis as a potential treatment strategy for cancer, the mechanisms underlying cancer cell insensitivity to cuproptosis remain poorly understood. In this study, we identified lactylated NUDT21 as a key driver of cuproptosis resistance in ESCC. Mechanistically, lactylation of NUDT21 induces an APA switch that reduces FDX1 levels, a critical protein involved in reducing Cu^2+^ to Cu^1+^ in mitochondria and facilitating excessive protein lipoylation^18,20^. Further analysis using clinical samples and TCGA datasets revealed that tumors with elevated levels of both LDHA and NUDT21, as well as increased K23-lactylated NUDT21, exhibit lower FDX1 levels, substantiating the role of the lactate-NUDT21-FDX1 axis in ESCC. Notably, a phase III clinical trial identified that melanoma patients with lower serum LDH levels showed improved progression-free survival when treated with a combination of elesclomol and paclitaxel^54^. Moreover, unmethylated NUDT21 has been implicated in cuproptosis insensitivity in prostate cancer by promoting copper export and inhibiting docosahexaenoic acid biosynthesis^55^. These findings collectively underscore the critical role of lactate and NUDT21 in mediating cuproptosis resistance. In line with this, our data revealed a strong synergistic effect between the clinically available LDHA inhibitor stiripentol and elesclomol in ESCC. Given the elevated serum copper levels in cancer patients, particularly in ESCC^21^, targeting the lactate-NUDT21-FDX1-cuproptosis axis may represent a promising therapeutic strategy for cancer treatment.

In summary, our findings highlight NUDT21 K23 lactylation as a pivotal modification that links l-lactate metabolism to APA regulation, enhancing dPAS usage in *FDX1* and resistance to cuproptosis (Fig. 6j). Inspiringly, the combination of stiripentol and elesclomol exerts a strong suppressive effect on ESCC, suggesting that targeting NUDT21, AARS1, or LDHA alongside elesclomol may represent a promising therapeutic strategy for ESCC treatment.

## Materials and methods

### Cell culture

All cell lines used in this study were sourced from the American Type Culture Collection (ATCC) and maintained under standardized conditions in a 5% CO_2_ incubator (Thermo Fisher Scientific) at 37 °C with humidified sterile water. The ESCC cell lines KYSE30 (RRID: CVCL_1351) and TE-1 (RRID: CVCL_1759) were cultured in Roswell Park Memorial Institute (RPMI) 1640 medium (C11875500BT, Gibco) supplemented with 10% fetal bovine serum (FBS) (FSP500, ExCell Bio). HEK293T (RRID: CVCL_0063) cells were maintained in Dulbecco’s modified Eagle’s medium (DMEM) (C11995500BT, Gibco) supplemented with 10% FBS. Both RPMI 1640 and DMEM media were routinely supplemented with Penicillin-Streptomycin Solution (BL505A, Biosharp). To preserve cell viability and functionality, resuscitated cells were not cultured for more than 2 months in vitro.

### Patients and samples

A total of 251 primary ESCC tissues were collected from patients who underwent surgical resection at the Sun Yat-sen University Cancer Center in Guangzhou between May 2005 and December 2018. Informed consent was obtained from all patients before sample collection. These samples were utilized to assess the protein levels of K23-lactylated NUDT21, NUDT21, LDHA, and FDX1. All cases included in this study had clear pathological diagnoses and had not received any local or systemic treatment prior to tissue collection. The Institutional Review Board of Sun Yat-sen University Cancer Center approved this study (Number SL-B2024-760-01).

### Animal study

All animals were housed in individually ventilated cages at the Center of Experimental Animal of SYSUCC under controlled temperature and humidity conditions, with unrestricted access to food and water. The study was approved by the Sun Yat-sen University Animal Care and Use Committee (Number SL-B2024-760-01).

For the in vivo tumorigenesis model, 2 ×10^6^ KYSE30 cells resuspended in 100 μL serum-free RPMI 1640 medium were subcutaneously injected into the flanks of 4-week-old nude mice (6 mice per group) (Vital River Laboratories, RRID: IMSR_RJ: BALB-C-NUDE). When tumor volumes reached ~50 mm^3^, mice were randomly assigned to treatment groups. Intraperitoneal injections were administered with saline, elesclomol (25 mg/kg, three times a week) (HY-12040, MCE), stiripentol (150 mg/kg, daily for five consecutive days per week) (HY-103392, MCE), or elesclomol + stiripentol. For β-alanine treatment, drinking water was replaced with 1.2% β-alanine solution (A606168, Sangon Biotech), refreshed weekly. Additionally, mice were orally gavaged with 200 μL of 1.2% β-alanine solution thrice weekly. Tumor growth was monitored every three days, and tumor volume was calculated using the following formula: volume (mm^3^) = (width (mm))^2^ × length (mm)/2.

### APA analysis

The PDUI values for TCGA ESCC patients (*n* = 95) were obtained from The Cancer 3′ UTR Atlas (TC3A) database^56^. Samples were stratified into *LDHA*^*high*^ and *LDHA*^*low*^ groups based on the median expression level of *LDHA*. For each gene, the PDUI values were averaged within the two groups, and then the overall changes in mean PDUI between the groups were compared.

### Western blot (WB)

Cells were pre-treated with l-lactate (71718, Sigma-Aldrich), d-lactate (71716, Sigma-Aldrich), oxamate (HY-W013032A, MCE), β-alanine, or acetate (S5636, Sigma-Aldrich) for 24 h before harvest. For protein extraction, IP lysis buffer (50 mM Tris-HCl, pH 7.4, 150 mM NaCl, 1 mM EDTA, 0.5% NP-40, and 10% glycerol) supplemented with protease inhibitor cocktail (CW2200S, CWBIO) was used to lyse cells on ice for 30 min. The supernatant was collected after centrifugation, and the protein concentration was quantified using the bicinchoninic acid assay kit (GK10009, GLPBIO). Denatured proteins were separated by 7.5%–12.5% SDS-PAGE gels and then transferred to a polyvinylidene fluoride membrane (3010040001, Roche). The membranes were incubated with the respective antibody at 4 °C overnight after blocking, then visualized by ChemiDoc Touch (Bio-Rad, RRID:SCR_021693). The antibodies used in this study are listed in Supplementary Table S1. The polyclonal antibody recognizing NUDT21 K23 lactylation (NUDT21-K23la) was generated using a K23-lactylated NUDT21 peptide TQFGNK^lac^YIQQTKP.

### RT-qPCR

Total RNA was extracted from the indicated cells using TRIzol (15596018, Invitrogen) following the manufacturer’s protocol. Subsequently, 1 μg of RNA was used to obtain cDNA by reverse transcription using PrimeScript™ RT reagent Kit (RR036A, TAKARA). According to the manufacturer’s instructions, qPCR was performed using 2× Color SYBR Green qPCR Master Mix (ROX2 plus) (A0012-R2, EZBioscience). For the analysis of mRNA 3′ UTR usage, two primer sets were designed: one pair targeted the CDS regions to represent the total transcripts, while the other pair targeted sequences immediately upstream of the dPAS to quantify the long transcripts. The primer sequences are listed in Supplementary Table S2.

### siRNA/shRNA design and construction

siRNAs used in this study were synthesized by RiboBio (Guangzhou, China). The shRNAs in the psi-LVRU6P vector specifically against NUDT21 were synthesized by FulenGen (Guangzhou, China). The shRNAs in the pLKO vector specifically against *FDX1* were synthesized by Tsingke (Guangzhou, China). The sequences of siRNAs and shRNAs were listed in Supplementary Table S3.

### IP

Equal amounts of cell lysates were incubated with protein A/G magnetic beads (HY-K0202, MCE) at 4 °C for 1 h to pre-clear non-specific proteins. Then discarded the beads and added the pre-washed new protein A/G magnetic beads, either mixed with specific antibodies or pre-coated with anti-Flag (B26102, Bimake), anti-Myc (B26302, Bimake), or anti-HA (B26201, Bimake). The mixture was incubated overnight at 4 °C on a rotator. Washed the beads using pre-chilled IP lysis buffer for 10 min at 4 °C and repeated 4 times. Discarded the IP lysis buffer and denatured the samples in 1× SDS buffer for further WB analysis.

### Pull-down assay and CBB staining

For pull-down assays involving interactions between NUDT21 and AARS1 or NUDT21 and HDAC2, 20 μg of purified recombinant proteins, including NUDT21-Flag, AARS1-HA, HDAC-Myc, or control peptides, were incubated with 15 μL pre-washed anti-Flag, anti-HA, or anti-Myc pre-coated beads in IP lysis buffer at 4 °C for 4 h. We washed the beads five times with IP lysis buffer and incubated with 20 μg of another recombinant protein at 4 °C overnight. The beads were washed five times the next day and then denatured in 1× SDS for further SDS-PAGE analyses. After electrophoresis, the gels were washed in double-distilled water and stained using Coomassie blue (PT0018, Leagene) for a minimum of 4 h. Excess and non-specific staining were removed by elution buffer (10% acetic acid, 40% methanol, and 50% double-distilled water).

### PAS-seq analysis

For each sample, 5 μg of total RNA was treated with RQ1 DNase (M6101, Promega) to remove DNA, fragmented RNAs were used for PAS-seq library preparation. mRNAs were captured with mRNA Capture Beads kit (N401, Vazyme). Fragmented mRNAs were used for directional PAS-seq library preparation by KAPA Stranded mRNA-Seq Kit for Illumina® Platforms (KK8544, Roche). Fragmented mRNAs were converted into double-stranded cDNA. Following end repair and A tailing, the DNAs were ligated to Diluted Roche Adaptor (KK8726, Roche). After purification of the ligation product and size fractioning to 300–500 bp, the ligated products were amplified and purified, quantified, and stored at –80 °C before sequencing. The strand marked with dUTP (the 2nd cDNA strand) is not amplified, allowing strand-specific sequencing. For high-throughput sequencing, the libraries were subjected to 150 nt paired-end sequencing on the Illumina Novaseq Xplus system. Raw reads containing more than 3-N bases were discarded. Adaptors and low-quality bases were trimmed using FASTX-Toolkit (Version 0.0.13), and reads less than 16 nt were removed. Processed reads were then aligned to the GRCh38 using HISAT2.

For PAS-seq data analysis, CAGEr was employed to identify and statistically assess PAS. TPM values were calculated for each PAS, and those with TPM < 1 were excluded. PAS within 24 nt were clustered into PAS clusters (PACs). PACs containing a single PAS with TPM < 3 were removed. PACs detected across all samples with TPM > 5 and within 500 nt of each other were further clustered. A shifting score was calculated for each PAC to quantify differences in the cumulative distribution of poly(A) signal along the 3′ UTR between samples. A positive shifting score indicated a shift in transcription termination outside the PAS region of the lower-expressing sample, whereas negative values suggested no spatial separation. Statistical significance was determined using the Kolmogorov-Smirnov test. Genes exhibiting a shifting score > 0, FDR ≤ 0.01, and TPM > 5 were considered to exhibit significant APA alterations.

### LC-MS/MS analysis

LC-MS/MS analysis was performed using MAGicomicomicS-MMB8X by QLBIO (Beijing, China). All RAW files were analyzed using the Proteome Discoverer suite (version 2.4, Thermo Fisher Scientific). MS2 spectra were searched against the UniProtKB human proteome database (RRID: SCR_002380).

### Protein expression and purification

Human *NUDT21*, *AARS1*, *HDAC2*, or *CPSF6* genes were cloned into the pET-28a vector, and the recombinant plasmids were expressed in *Escherichia coli* BL21 (DE3) in Luria-Bertani medium containing 0.1 mg/mL ampicillin and 34 μg/mL chloromycetin. Protein expression was induced with 0.5 mM IPTG when the optical density at 600 nm reached 0.6–0.8. The cells were then cultured at 15 °C overnight. After incubation, cells were harvested and lysed using lysis buffer (10 mM imidazole, 50 mM NaH_2_PO_4_, 1 mM PMSF, 300 mM NaCl, pH 8.0). The lysates were centrifuged at 18,000 rpm for 1 h. Pre-equilibrate the Ni-NTA column with lysis buffer and load the cell supernatant into the column after being filtered by a 0.45-μm strainer. Washed the column with wash buffer (lysis buffer with 30 mM imidazole) and then eluted the purified proteins with elution buffer (lysis buffer supplemented with 300 mM imidazole). The purified proteins were further lyophilized by ALPHA1-2LD plus (Christ, Germany) and dissolved in PBS. The purity and size of the proteins were confirmed by SDS-PAGE analysis.

### In vitro latcylation assays

Purified recombinant NUDT21 (10 μg) was incubated with 10 μg of recombinant AARS1 in the presence or absence of l-lactate in a 25 μL reaction mixture containing 50 mM HEPES (pH 7.5), 25 mM KCl, 2 mM MgCl_2_, 2 mM lactate, and 4 mM ATP. The reaction was conducted at 37 °C for 1 h. The reactions were terminated by the addition of 5× SDS loading buffer. Lactylation of NUDT21 was detected by WB using an anti-Klac antibody.

### PLA

The interaction between NUDT21 and CPSF6 was assessed by DUOLINK® PLA (DUO92102, Sigma-Aldrich) according to the manufacturer’s instructions. In brief, cells were first blocked and then incubated with anti-Flag (F1804, Sigma-Aldrich) at a 1:200 dilution and anti-CPSF6 (15489-1-AP, Proteintech) at a 1:200 dilution at 4 °C overnight. Two PLA probes were used to bind the primary antibodies at 37 °C for 1 h. Subsequently, the Ligase and Polymerase were added, and incubation was carried out at 37 °C for 30 min and 100 min, respectively. Next, the cells were washed using Wash Buffer A and B. The cells were mounted with Duolink In Situ Mounting Medium with DAPI, followed by immunofluorescence analysis.

### RIP

The interaction between NUDT21 and *FDX1* RNA was evaluated using Magna RIP^TM^ Kit (17-700, Sigma-Aldrich) following the manufacturer’s instructions. Briefly, cells were lysed in RIP lysis buffer supplemented with protease and RNase inhibitors. After centrifugation, supernatants were incubated with magnetic beads pre-conjugated with anti-NUDT21 or control IgG antibodies at 4 °C overnight. Following incubation, the beads were washed thoroughly and treated with proteinase K-containing buffer to digest protein complexes. The supernatants were collected, and RNA was extracted using TRIzol. The purified RNA was subsequently analyzed by RT-qPCR.

### Copper content detection

Copper levels in xenograft tumors were quantified using the Copper Microplate Assay Kit (abs580140, Absin) following the manufacturer’s instructions. Absorbance was measured at 605 nm, and copper concentration was normalized to tumor weight.

### Dual-luciferase reporter assay

The long 3′ UTR and the short 3′ UTR of *FDX1* were subcloned into the pmirGLO vector. To obtain expression of only the long 3′ UTR of *FDX1*, the proximal poly(A) signal sequence was mutated from AUUAAA to CCCCCC. After transfection for 48 h, relative luciferase activity was detected with a Dual-Luciferase Reporter Assay System (E1910, Promega) according to the user’s manual. *Renilla* luciferase activity was detected and used to normalize Firefly luciferase activity.

### IHC

The paraffin tissue sections were first baked at 56 °C for 30 min and then subjected to dewaxing in dimethylbenzene and rehydration with graded ethanol. Antigen retrieval of the sections was performed using high-pressure and heat repair. After natural cooling, the sections were incubated with 3% hydrogen peroxide for 10 min to inactivate the endogenous peroxidase. Subsequently, the sections were blocked with 1% BSA at 37 °C for 1 h and incubated with an anti-NUDT21 (1:200), anti-LDHA (1:200), anti-FDX1 (1:200), or anti-NUDT21-K23la (1:100) at 4 °C overnight. A secondary antibody (PV6000, Zsbio) was used to bind the primary antibody at 37 °C for 1 h, and DAB (ZLI9017, Zsbio) was used to stain the target protein, followed by hematoxylin staining.

### Dot blot

Synthetic peptides corresponding to NUDT21 K23 (TQFGNKYIQQTKP) and K23la (TQFGNK^lac^YIQQTKP) were prepared at a concentration of 2 mg/mL. The peptides were spotted onto a nitrocellulose membrane at varying amounts (10, 50, and 100 ng). After air-drying, the membrane was blocked with 5% nonfat milk in TBST buffer before proceeding with immunoblot analysis.

### Statistical analysis

Statistical analyses were conducted using GraphPad Prism (Version 8; La Jolla, CA, USA, RRID: SCR_002798) or SPSS (Version 25.0; Armonk, NY, USA, RRID: SCR_016479). The Kaplan–Meier method was used to calculate the cumulative overall survival data, and the log-rank test was used for analysis. The correlations between *NUDT21*, *LDHA*, *AARS1*, *HDAC2*, and *FDX1* were evaluated by Pearson correlation analysis. For comparisons between the two groups, two-tailed Student’s *t*-test and Mann–Whitney U test were used. ANOVA was used for multiple comparisons among more than two groups. The Kaplan–Meier method was used to calculate the cumulative overall survival data, and the log-rank test was used for analysis. Data were shown as mean ± SD, and *P* < 0.05 was considered statistically significant.

## Supplementary information

Supplementary Figs. 1-10 and Tables S1-3.

## Data Availability

PAS-seq data have been deposited in the Gene Expression Omnibus under a accession number: GSE284372. LC-MS/MS raw data are available at the iProX with the identifier PXD041430.

## References

[1] Mitschka, S. & Mayr, C. Context-specific regulation and function of mRNA alternative polyadenylation. *Nat. Rev. Mol. Cell Biol.* **23**, 779–796 (2022). 35798852 10.1038/s41580-022-00507-5 PMC9261900

[2] Mohanan, N. K., Shaji, F., Koshre, G. R. & Laishram, R. S. Alternative polyadenylation: An enigma of transcript length variation in health and disease. *Wiley Interdiscip. Rev. RNA* **13**, e1692 (2022). 34581021 10.1002/wrna.1692

[3] Subramanian, A. et al. Alternative polyadenylation is a determinant of oncogenic Ras function. *Sci. Adv.* **7**, eabh0562 (2021). 34919436 10.1126/sciadv.abh0562 PMC8682989

[4] Zhang, N. et al. LINC00921 reduces lung cancer radiosensitivity by destabilizing NUDT21 and driving aberrant MED23 alternative polyadenylation. *Cell Rep.* **42**, 113479 (2023). 37999979 10.1016/j.celrep.2023.113479

[5] Tan, Y. et al. Alternative polyadenylation reprogramming of MORC2 induced by NUDT21 loss promotes KIRC carcinogenesis. *JCI Insight* **8**, e162893 (2023). 10.1172/jci.insight.162893 PMC10561724 37737260

[6] Park, H. J. et al. 3’ UTR shortening represses tumor-suppressor genes in trans by disrupting ceRNA crosstalk. *Nat. Genet.* **50**, 783–789 (2018). 29785014 10.1038/s41588-018-0118-8 PMC6689271

[7] Chen, X. et al. CSTF2-induced shortening of the RAC1 3’UTR promotes the pathogenesis of urothelial carcinoma of the bladder. *Cancer Res.* **78**, 5848–5862 (2018). 30143523 10.1158/0008-5472.CAN-18-0822

[8] Yang, S. W. et al. A Cancer-specific ubiquitin ligase drives mRNA alternative polyadenylation by ubiquitinating the mRNA 3’ end processing complex. *Mol. Cell* **77**, 1206–1221.e7 (2020). 31980388 10.1016/j.molcel.2019.12.022

[9] Thivierge, C. et al. Alternative polyadenylation confers Pten mRNAs stability and resistance to microRNAs. *Nucleic Acids Res.* **46**, 10340–10352 (2018). 30053103 10.1093/nar/gky666 PMC6212768

[10] Lin, P. et al. RBBP6 maintains glioblastoma stem cells through CPSF3-dependent alternative polyadenylation. *Cell Discov.* **10**, 32 (2024). 38503731 10.1038/s41421-024-00654-3 PMC10951364

[11] Liberti, M. V. & Locasale, J. W. The Warburg effect: How does it benefit cancer cells?. *Trends Biochem. Sci.* **41**, 211–218 (2016). 26778478 10.1016/j.tibs.2015.12.001 PMC4783224

[12] Li, X. et al. Lactate metabolism in human health and disease. *Signal Transduct. Target. Ther.* **7**, 305 (2022). 10.1038/s41392-022-01151-3 PMC9434547 36050306

[13] Chen, Y. et al. Metabolic regulation of homologous recombination repair by MRE11 lactylation. *Cell* **187**, 294–311.e21 (2024). 38128537 10.1016/j.cell.2023.11.022 PMC11725302

[14] Zhang, D. et al. Metabolic regulation of gene expression by histone lactylation. *Nature* **574**, 575–580 (2019). 31645732 10.1038/s41586-019-1678-1 PMC6818755

[15] Chen, H. et al. NBS1 lactylation is required for efficient DNA repair and chemotherapy resistance. *Nature* **631**, 663–669 (2024). 38961290 10.1038/s41586-024-07620-9 PMC11254748

[16] Li, H. et al. AARS1 and AARS2 sense l-lactate to regulate cGAS as global lysine lactyltransferases. *Nature* **634**, 1229–1237 (2024). 39322678 10.1038/s41586-024-07992-y

[17] Tong, H. et al. Dual impacts of serine/glycine-free diet in enhancing antitumor immunity and promoting evasion via PD-L1 lactylation. *Cell Metab.* **36**, 2493–2510.e9 (2024). 39577415 10.1016/j.cmet.2024.10.019

[18] Tang, D., Kroemer, G. & Kang, R. Targeting cuproplasia and cuproptosis in cancer. *Nat. Rev. Clin. Oncol.* **21**, 370–388 (2024). 38486054 10.1038/s41571-024-00876-0

[19] Tsvetkov, P. et al. Copper induces cell death by targeting lipoylated TCA cycle proteins. *Science* **375**, 1254–1261 (2022). 35298263 10.1126/science.abf0529 PMC9273333

[20] Tsvetkov, P. et al. Mitochondrial metabolism promotes adaptation to proteotoxic stress. *Nat. Chem. Biol.* **15**, 681–689 (2019). 31133756 10.1038/s41589-019-0291-9 PMC8183600

[21] Mir, M. M. et al. Studies on association between Copper excess, Zinc deficiency and TP53 mutations in esophageal squamous cell carcinoma from Kashmir valley, India-A high risk area. *Int. J. Health Sci.* **1**, 35–42 (2007). PMC3068648 21475450

[22] Sun, L. et al. Lactylation of METTL16 promotes cuproptosis via m(6)A-modification on FDX1 mRNA in gastric cancer. *Nat. Commun.* **14**, 6523 (2023). 37863889 10.1038/s41467-023-42025-8 PMC10589265

[23] Shanbhag, V. C. et al. Copper metabolism as a unique vulnerability in cancer. *Biochim. Biophy. Acta Mol. Cell Res.* **1868**, 118893 (2021). 10.1016/j.bbamcr.2020.118893 PMC7779655 33091507

[24] Valvona, C. J., Fillmore, H. L., Nunn, P. B. & Pilkington, G. J. The regulation and function of lactate dehydrogenase A: Therapeutic potential in brain tumor. *Brain Pathol.* **26**, 3–17 (2016). 26269128 10.1111/bpa.12299 PMC8029296

[25] Moreno-Yruela, C., Bæk, M., Monda, F. & Olsen, C. A. Chiral posttranslational modification to lysine ε-amino groups. *Acc. Chem. Res.* **55**, 1456–1466 (2022). 35500056 10.1021/acs.accounts.2c00115

[26] Zhang, D. et al. Lysine L-lactylation is the dominant lactylation isomer induced by glycolysis. *Nat. Chem. Biol.* **21**, 91–99 (2024). 39030363 10.1038/s41589-024-01680-8 PMC11666458

[27] Zhu, C. et al. CPSF6-mediated XBP1 3’UTR shortening attenuates cisplatin-induced ER stress and elevates chemo-resistance in lung adenocarcinoma. *Drug Resist. Updat.* **68**, 100933 (2023). 36821972 10.1016/j.drup.2023.100933

[28] Cai, X. et al. Lactate activates the mitochondrial electron transport chain independently of its metabolism. *Mol. Cell* **83**, 3904–3920.e7 (2023). 37879334 10.1016/j.molcel.2023.09.034 PMC10752619

[29] Duan, Y. et al. Integrated lactylome characterization reveals the molecular dynamics of protein regulation in gastrointestinal cancers. *Adv. Sci.* **11**, e2400227 (2024). 10.1002/advs.202400227 PMC11425215 39018247

[30] Yang, D. et al. Identification of lysine-lactylated substrates in gastric cancer cells. *iScience* **25**, 104630 (2022). 35800753 10.1016/j.isci.2022.104630 PMC9253728

[31] Yang, Z. et al. Lactylome analysis suggests lactylation-dependent mechanisms of metabolic adaptation in hepatocellular carcinoma. *Nat. Metab.* **5**, 61–79 (2023). 36593272 10.1038/s42255-022-00710-w

[32] Moreno-Yruela, C. et al. Class I histone deacetylases (HDAC1-3) are histone lysine delactylases. *Sci. Adv.* **8**, eabi6696 (2022). 35044827 10.1126/sciadv.abi6696 PMC8769552

[33] Zong, Z. et al. Alanyl-tRNA synthetase, AARS1, is a lactate sensor and lactyltransferase that lactylates p53 and contributes to tumorigenesis. *Cell* **187**, 2375–2392.e33 (2024). 38653238 10.1016/j.cell.2024.04.002

[34] Masamha, C. P. The emerging roles of CFIm25 (NUDT21/CPSF5) in human biology and disease. *Wiley Interdiscip. Rev. RNA* **14**, e1757 (2023). 35965101 10.1002/wrna.1757 PMC9925614

[35] Brumbaugh, J. et al. Nudt21 controls cell fate by connecting alternative polyadenylation to chromatin signaling. *Cell* **172**, 106–120.e21 (2018). 29249356 10.1016/j.cell.2017.11.023 PMC5766360

[36] Shimazu, T., Horinouchi, S. & Yoshida, M. Multiple histone deacetylases and the CREB-binding protein regulate pre-mRNA 3’-end processing. *J. Biol. Chem.* **282**, 4470–4478 (2007). 17172643 10.1074/jbc.M609745200

[37] Rho, H., Terry, A. R., Chronis, C. & Hay, N. Hexokinase 2-mediated gene expression via histone lactylation is required for hepatic stellate cell activation and liver fibrosis. *Cell Metab.* **35**, 1406–1423.e8 (2023). 37463576 10.1016/j.cmet.2023.06.013 PMC11748916

[38] Dettwiler, S., Aringhieri, C., Cardinale, S., Keller, W. & Barabino, S. M. Distinct sequence motifs within the 68-kDa subunit of cleavage factor Im mediate RNA binding, protein-protein interactions, and subcellular localization. *J. Biol. Chem.* **279**, 35788–35797 (2004). 15169763 10.1074/jbc.M403927200

[39] Martin, G., Gruber, A. R., Keller, W. & Zavolan, M. Genome-wide analysis of pre-mRNA 3’ end processing reveals a decisive role of human cleavage factor I in the regulation of 3’ UTR length. *Cell Rep.* **1**, 753–763 (2012). 22813749 10.1016/j.celrep.2012.05.003

[40] Khan, F. et al. Lactate dehydrogenase A regulates tumor-macrophage symbiosis to promote glioblastoma progression. *Nat. Commun.* **15**, 1987 (2024). 38443336 10.1038/s41467-024-46193-z PMC10914854

[41] Vander Heiden, M. G., Cantley, L. C. & Thompson, C. B. Understanding the Warburg effect: the metabolic requirements of cell proliferation. *Science* **324**, 1029–1033 (2009). 19460998 10.1126/science.1160809 PMC2849637

[42] Ju, J. et al. The alanyl-tRNA synthetase AARS1 moonlights as a lactyltransferase to promote YAP signaling in gastric cancer. *J. Clin. Invest.* **134**, e174587 (2024). 38512451 10.1172/JCI174587 PMC11093599

[43] Gaffney, D. O. et al. Non-enzymatic lysine lactoylation of glycolytic enzymes. *Cell Chem. Biol.* **27**, 206–213.e6 (2020). 31767537 10.1016/j.chembiol.2019.11.005 PMC7395678

[44] Jennings, E. Q. et al. Sirtuin 2 regulates protein lactoylLys modifications. *Chembiochem* **22**, 2102–2106 (2021). 33725370 10.1002/cbic.202000883 PMC8205944

[45] Lv, M. et al. CDK7-YAP-LDHD axis promotes d-lactate elimination and ferroptosis defense to support cancer stem cell-like properties. *Signal Transduct. Target. Ther.* **8**, 302 (2023). 10.1038/s41392-023-01555-9 PMC10427695 37582812

[46] Yang, Q., Gilmartin, G. M. & Doublié, S. Structural basis of UGUA recognition by the Nudix protein CFI(m)25 and implications for a regulatory role in mRNA 3’ processing. *Proc. Natl. Acad. Sci. USA* **107**, 10062–10067 (2010). 10.1073/pnas.1000848107 PMC2890493 20479262

[47] Zhu, Y. et al. Molecular mechanisms for CFIm-mediated regulation of mRNA alternative polyadenylation. *Mol. Cell* **69**, 62–74.e4 (2018). 29276085 10.1016/j.molcel.2017.11.031 PMC5756121

[48] Xing, Y. et al. Downregulation of NUDT21 contributes to cervical cancer progression through alternative polyadenylation. *Oncogene* **40**, 2051–2064 (2021). 33619322 10.1038/s41388-021-01693-w

[49] Sun, M. et al. NUDT21 regulates 3’-UTR length and microRNA-mediated gene silencing in hepatocellular carcinoma. *Cancer Lett.* **410**, 158–168 (2017). 28964783 10.1016/j.canlet.2017.09.026

[50] Xiong, M. et al. NUDT21 inhibits bladder cancer progression through ANXA2 and LIMK2 by alternative polyadenylation. *Theranostics* **9**, 7156–7167 (2019). 31695759 10.7150/thno.36030 PMC6831288

[51] Liu, K. et al. CFIm25-regulated lncRNA acv3UTR promotes gastric tumorigenesis via miR-590-5p/YAP1 axis. *Oncogene* **39**, 3075–3088 (2020). 32066878 10.1038/s41388-020-1213-8 PMC7142022

[52] Zhou, Y. et al. Nudt21-mediated alternative polyadenylation of MZT1 3’UTR contributes to pancreatic cancer progression. *iScience* **27**, 108822 (2024). 38303721 10.1016/j.isci.2024.108822 PMC10831950

[53] Liu, W. et al. Pan-cancer analysis of NUDT21 and its effect on the proliferation of human head and neck squamous cell carcinoma. *Aging* **16**, 3363–3385 (2024). 38349866 10.18632/aging.205539 PMC10929839

[54] O’Day, S. J. et al. Final results of phase III SYMMETRY study: randomized, double-blind trial of elesclomol plus paclitaxel versus paclitaxel alone as treatment for chemotherapy-naive patients with advanced melanoma. *J. Clin. Oncol.* **31**, 1211–1218 (2013). 23401447 10.1200/JCO.2012.44.5585

[55] Lin, S. C. et al. Un-methylation of NUDT21 represses docosahexaenoic acid biosynthesis contributing to enzalutamide resistance in prostate cancer. *Drug Resist. Updat.* **77**, 101144 (2024). 39208673 10.1016/j.drup.2024.101144

[56] Feng, X., Li, L., Wagner, E. J. & Li, W. TC3A: the cancer 3’ UTR atlas. *Nucleic Acids Res.* **46**, D1027–D1030 (2018). 30053266 10.1093/nar/gkx892 PMC5753254

