# Supplementary material for: NUDT21 lactylation reprograms alternative polyadenylation to promote cuproptosis resistance
Source: Cell Discov. 2025 May 28;11:52. doi: 10.1038/s41421-025-00804-1 (PMC12116747; doi:10.1038/s41421-025-00804-1)
Supplement: Supplementary file 1 — Supplementary Figs. 1-10 and Tables S1-3. [file 41421_2025_804_MOESM1_ESM.pdf]

Supplementary Information for

NUDT21 lactylation reprograms alternative polyadenylation to promote cuproptosis resistance

Supplementary Figures

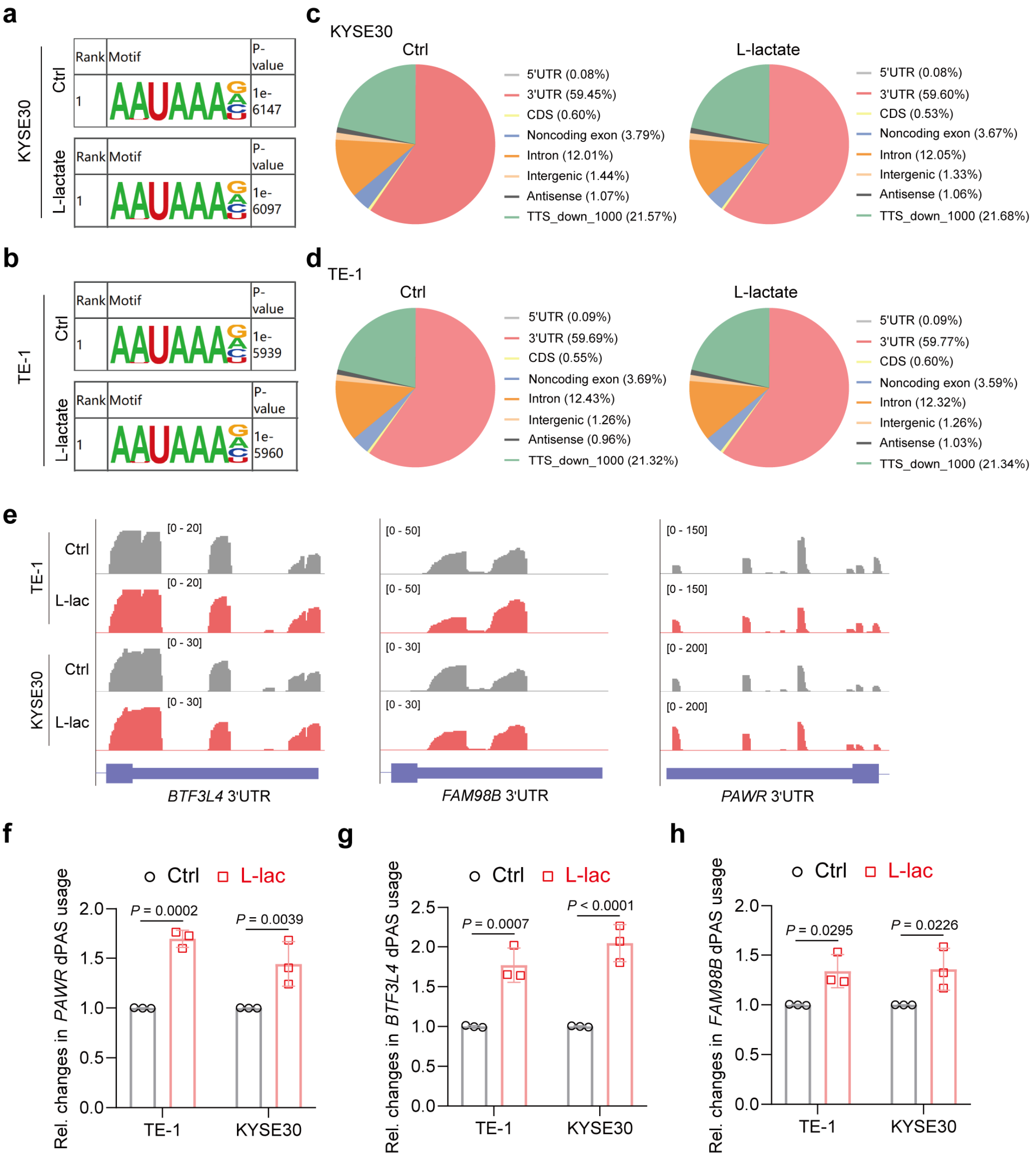

Fig. S1 L-lactate regulates APA switch in ESCC cells.

(**a, b**) The most enriched PAS motifs identified from PAS-seq analysis in KYSE30 and TE-1 cells.

(**c, d**) Pie charts illustrating the distribution of PASs from PAS-seq analysis in KYSE30 and TE-1 cells.

(**e**) PAS-seq density plots for *BTF3L4*, *FAM98B*, and *PAWR*, whose 3′ UTRs are lengthened in response to L-lactate.

(**f-h**) RT-qPCR analysis of dPAS usage for *BTF3L4*, *FAM98B*, and *PAWR* in ESCC cells treated with L-lactate (20 mM) for 24 h.

Data are presented as mean ± SD; *P* value was calculated by Student's *t* test (f-h).

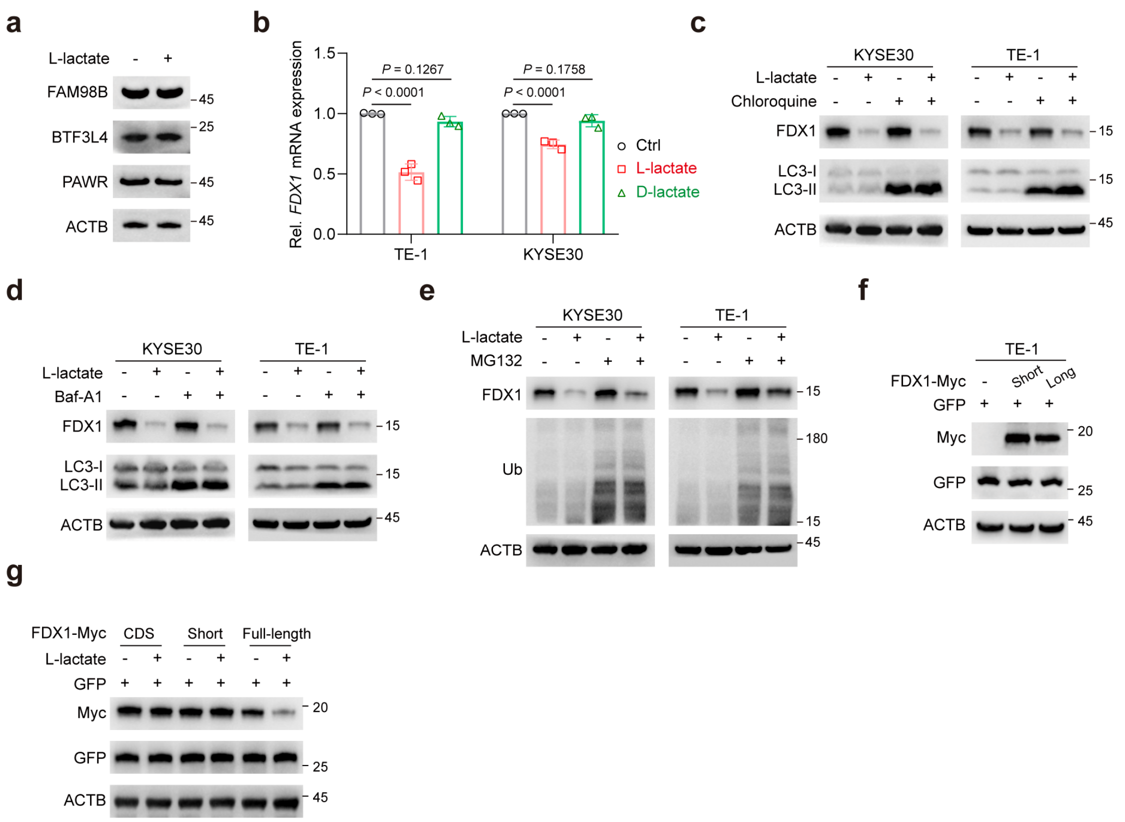

Fig. S2 L-lactate upregulates FDX1 protein levels via APA.

(**a**) Immunoblot analysis of BTF3L4, FAM98B, and PAWR protein levels in KYSE30 cells treated with L-lactate (20 mM) for 24 h.

(**b**) Relative mRNA expression of *FDX1* in ESCC cells treated with L-lactate (20 mM) or D-lactate (20 mM) for 24 h.

(**c-e**) Immunoblot analysis examining the effects of chloroquine (30 μM, 12 h), Baf-A1 (20 nM, 3 h), or MG132 (10 μM, 3 h) on the L-lactate-induced reduction of FDX1 protein levels.

(**f, g**) Immunoblot analysis of FDX1 and GFP protein levels in TE-1 cells transfected with the indicated plasmids.

Data are presented as mean ± SD; *P* value was calculated by Student's *t* test (b).

a

Lactylated targets within the 3′ end processing machinery

Study	Protein
Yang, 2023	CFSP1, CPSF6<br>NUDT21, FIP1L1<br>WDR33
Duan, 2024	CFSP1, CPSF2<br>CFSP3, CPSF6<br>NUDT21, FIP1L1<br>WDR33
Yang, 2024	CFSP1, CPSF2<br>NUDT21, FIP1L1<br>WDR33

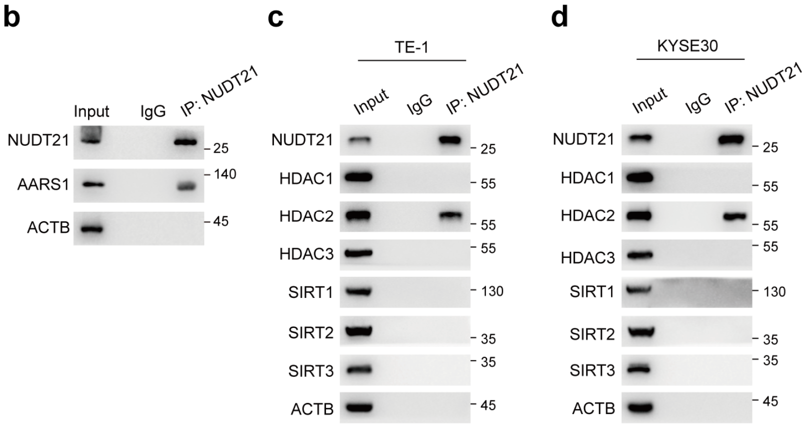

Fig. S3 NUDT21 lactylation is regulated by AARS1 and HDAC2.

(**a**) Table summarizing the lactylation of APA regulators identified by three integrated studies.

(**b**) The interaction between NUDT21 and AARS1 was detected by Co-IP in 293T cells.

(**c, d**) The interaction between NUDT21 and HDAC2 was detected by Co-IP in ESCC cells.

a

Lactylated sites within CPSF1

Study	Site	Score
Yang, 2023	K912	112.95
	K1326	111.39
Duan, 2024	K420	126.48
	K912	137.58
	K1326	130.31
Yang, 2024	K912	162.46

b

Lactylated sites within CPSF2

Study	Site	Score
Yang, 2023	N/A	N/A
Duan, 2024	K459	61.194
Yang, 2024	K584	48.423

c

Lactylated sites within CPSF3

Study	Site	Score
Yang, 2023	N/A	N/A
Duan, 2024	K600	49.325
Yang, 2024	N/A	N/A

d

Lactylated sites within CPSF6

Study	Site	Score
Yang, 2023	K79	92.707
	K185	93.576
Duan, 2024	K79	77.157
	K173	76.255
	K185	73.805
Yang, 2024	N/A	N/A

e

Lactylated sites within FIP1L1

Study	Site	Score
Yang, 2023	K135	52.591
	K235	105.81
Duan, 2024	K135	86.205
	K235	150.88
	K294	71.219
Yang, 2024	K135	63.624

f

Lactylated sites within WDR33

Study	Site	Score
Yang, 2023	K248	72.031
	K1023	66.913
Duan, 2024	K906	80.175
	K1023	78.673
	K1044	63.397
	K1161	65.879
	K1232	112.95
Yang, 2024	K956	77.221
	K1023	70.452
	K1161	51.135

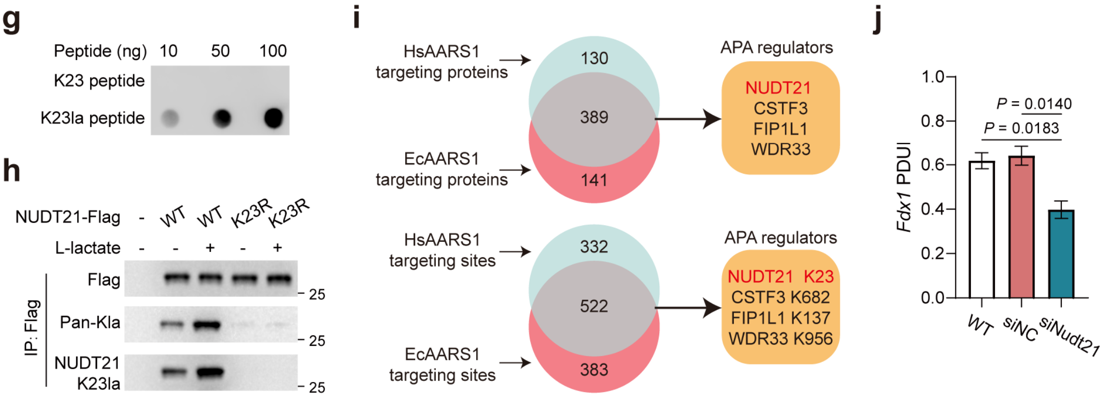

Fig. S4 NUDT21 is lactylated at K23.

(**a-f**) Table summarizing the lactylation sites of APA regulators identified by three integrated studies.

(**g**) Immunoblot analysis of synthetic peptides immobilized on a nitrocellulose membrane using an anti-NUDT21-K23la antibody.

(**h**) Immunoblot analysis of K23-lactylated NUDT21 levels in 293T cells transfected with NUDT21 WT or K23R mutant, with or without L-lactate (20 mM) stimulation for 24 h.

(**i**) Overlapping lactylated sites (lower) and proteins (upper) identified as targets of human AARS1 (HsAARS1) and *E. coli* AARS1 (EcAARS1).

(**j**) Analysis of the *Fdx1* PDUI in MEF cells following NUDT21 knockdown.

Data are presented as mean ± SD; *P* value was calculated by Student's *t* test (j).

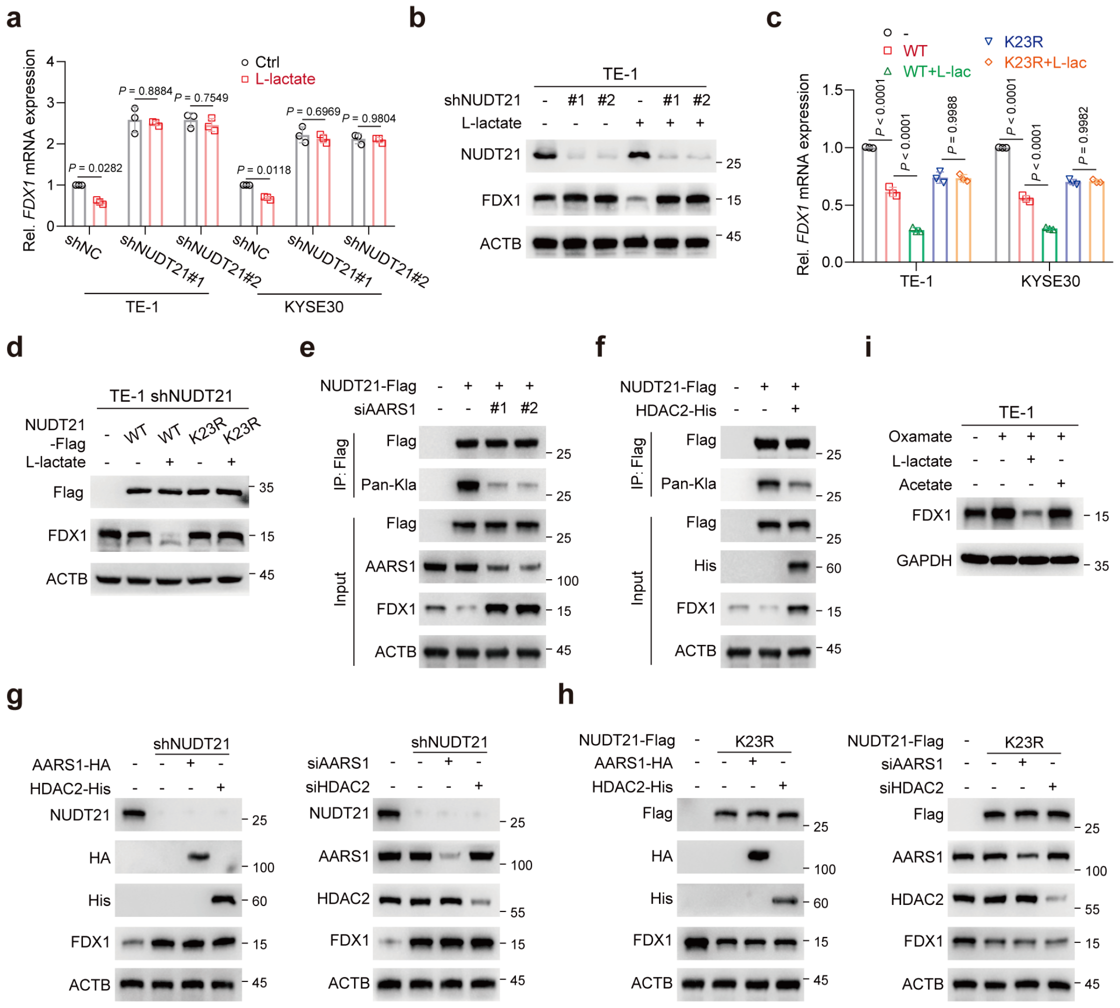

Fig. S5 NUDT21 lactylation upregulates FDX1 protein levels.

(**a**) Relative mRNA expression of *FDX1* in NUDT21 knockdown ESCC cells, with or without L-lactate (20 mM) stimulation for 24 h.

(**b**) Immunoblot analysis of FDX1 protein levels in TE-1 cells following NUDT21 knockdown, with or without L-lactate (20 mM) stimulation for 24 h.

(**c**) Relative mRNA expression of *FDX1* in NUDT21-knockdown ESCC cells transfected with NUDT21 WT or K23R mutant, with or without L-lactate (20 mM) stimulation for 24 h.

(**d**) Immunoblot analysis of FDX1 protein levels in NUDT21-knockdown TE-1 cells transfected with NUDT21 WT or K23R mutant, with or without L-lactate (20 mM) stimulation for 24 h.

(**e, f**) Immunoblot analysis of NUDT21 lactylation and FDX1 protein levels in KYSE30 cells transfected with AARS1-specific siRNAs (e) or HDAC2-Myc (f). Cells were pre-

treated with oxamate before harvest.

(**g, h**) Immunoblot analysis of FDX1 protein levels in NUDT21-knockdown (g) or NUDT21 K23R (h) KYSE30 cells transfected with the indicated plasmids or siRNAs.

(**i**) Immunoblot analysis of FDX1 protein levels in TE-1 cells treated with oxamate (20 mM), L-lactate (20 mM), or acetate (10 mM) for 24 h.

Data are presented as mean ± SD; *P* value was calculated by Student's *t* test (a and c).

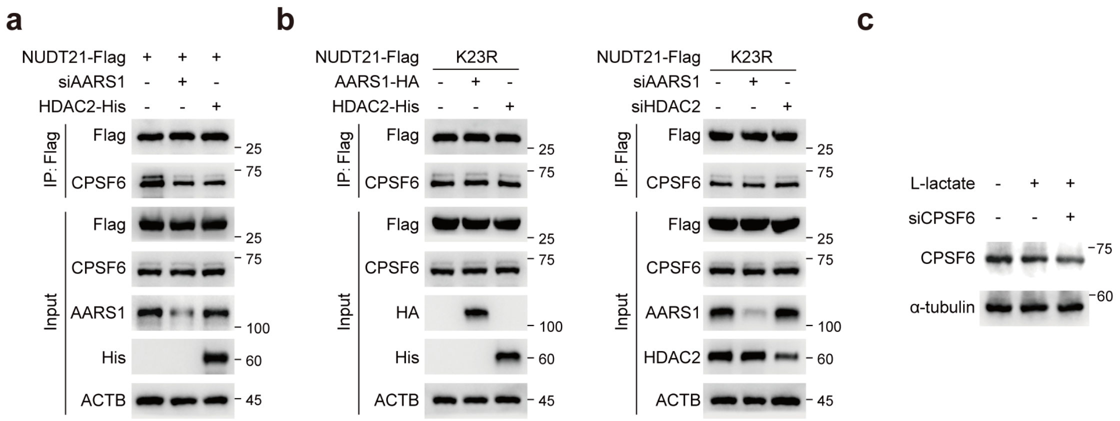

Fig. S6 NUDT21 lactylation enhances its interaction with CPSF6.

(**a**) Immunoblot analysis of the interaction between NUDT21 and CPSF6 in KYSE30 cells transfected with the indicated plasmids or siRNAs.

(**b**) Immunoblot analysis of the interaction between NUDT21 and CPSF6 in NUDT21 K23R cells transfected with the indicated plasmids or siRNAs.

(**c**) Immunoblot analysis of CPSF6 levels to test the silencing effect of CPSF6-specific siRNA in KYSE30 cells.

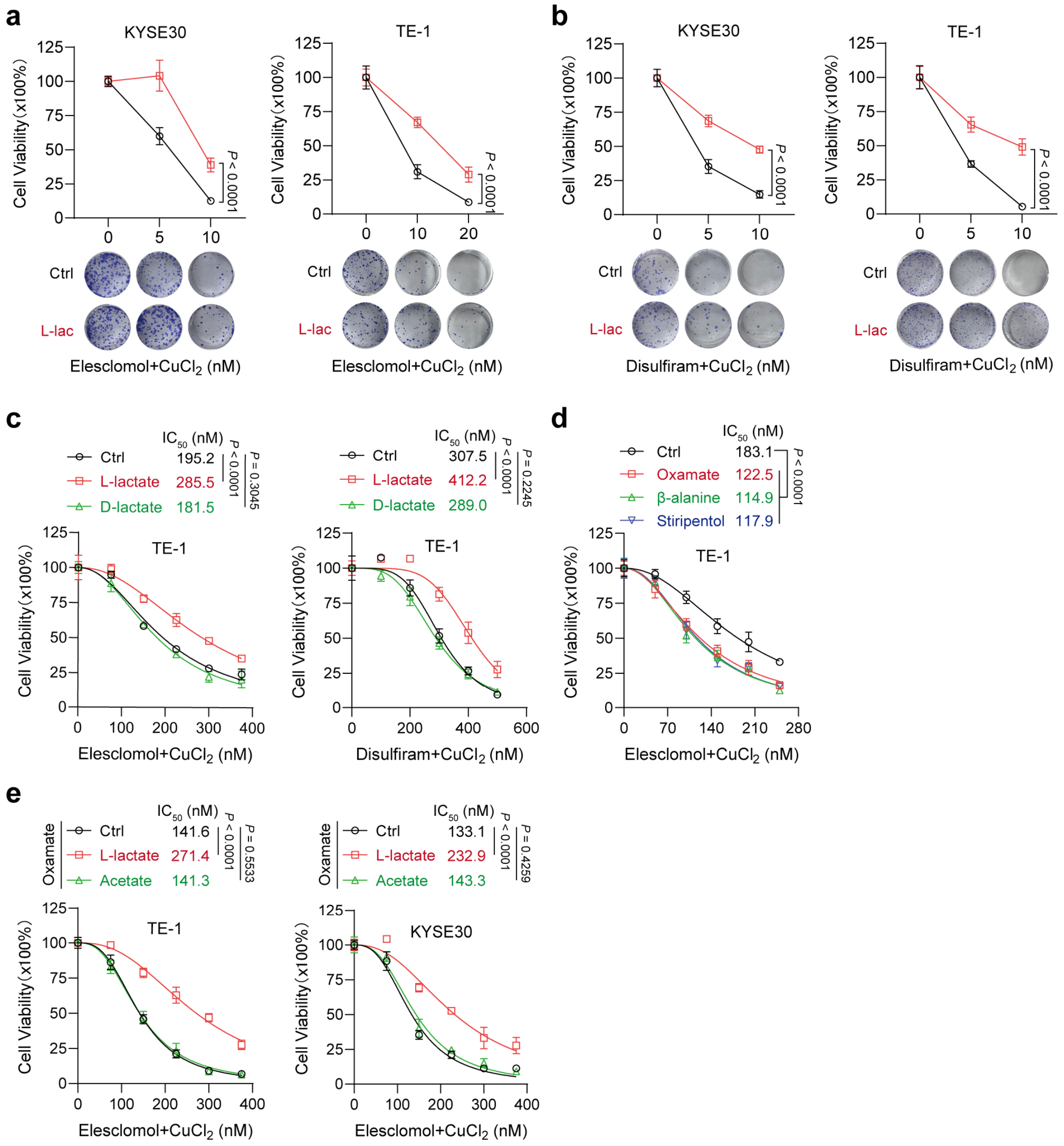

Fig. S7 L-lactate promotes resistance to cuproptosis in ESCC.

(**a, b**) The cell viability of L-lactate-treated KYESE30 and TE-1 cells in response to the indicated concentrations of elesclomol-Cu^{2+} or disulfiram-Cu^{2+}, as measured by colony formation assays.

(**c-e**) The cell viability of ESCC cells treated with L-lactate, D-lactate, oxamate, β-alanine, stiripentol, or acetate in response to elesclomol-Cu^{2+} or disulfiram-Cu^{2+}, as measured by CCK8.

Data are presented as mean ± SD; *P* value was calculated by two-way ANOVA (a-e).

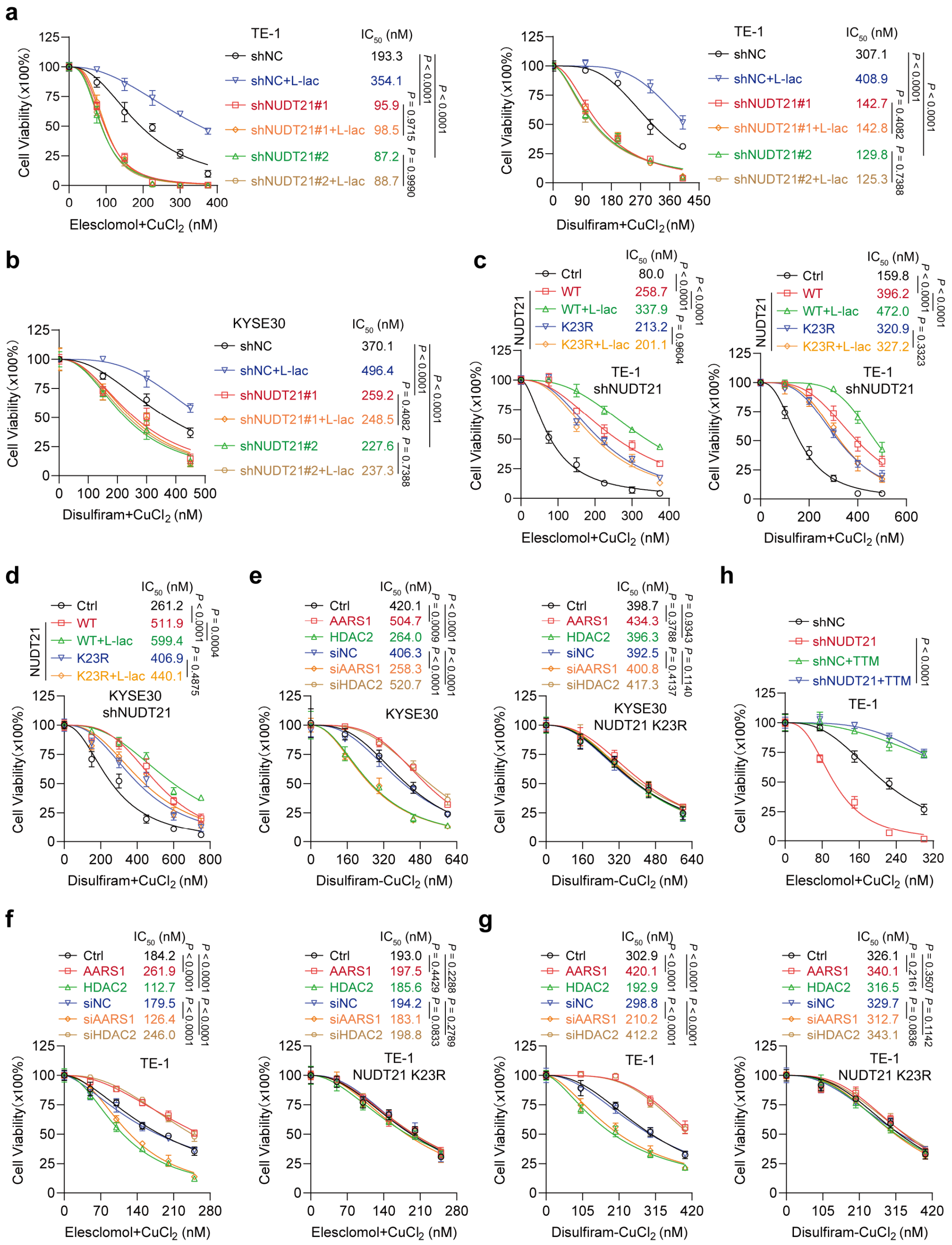

Fig. S8 NUDT21 plays a key role in L-lactate-induced cuproptosis resistance.

(**a, b**) The cell viability of NUDT21-knockdown ESCC cells following treatment with elesclomol-Cu^{2+} or disulfiram-Cu^{2+}, with or without L-lactate (20 mM), as measured by CCK8.

(**c, d**) The cell viability of NUDT21 WT- or K23R-overexpressing ESCC cells was measured by CCK8 after treatment with elesclomol-Cu^{2+} or disulfiram-Cu^{2+}, with or

without L-lactate (20 mM).

(**e-g**) The cell viability of ESCC cells transfected with the indicated plasmids or siRNAs in response to elesclomol-Cu^{2+} or disulfiram-Cu^{2+}, as measured by CCK8.

(**h**) TTM (1μM) treatment reversed the growth inhibition induced by NUDT21 knockdown in TE-1 cells exposed to elesclomol-Cu^{2+}.

Data are presented as mean ± SD; *P* value was calculated by two-way ANOVA (a-h).

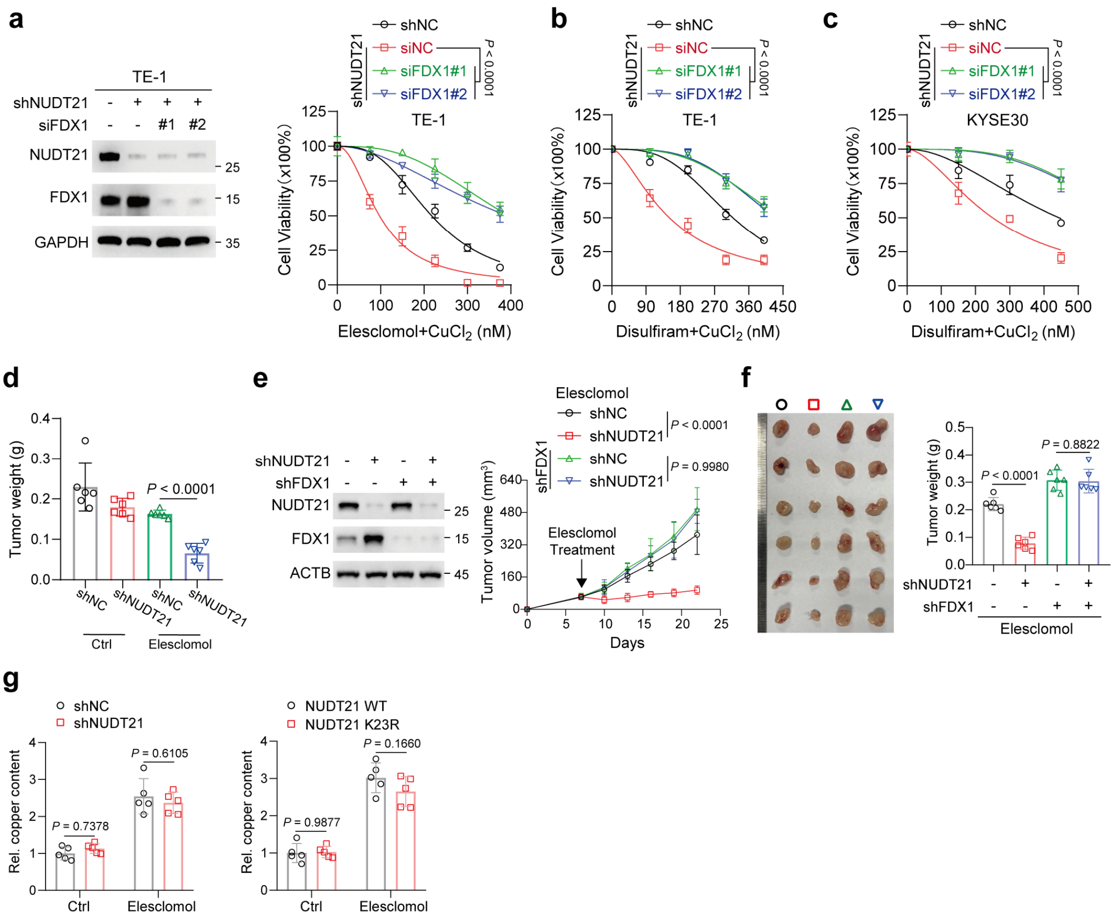

Fig. S9 NUDT21 mediates cuproptosis resistance via FDX1.

(**a-c**) FDX1 depletion reversed the growth inhibition induced by NUDT21 knockdown in ESCC cells treated with elesclomol-Cu^{2+} or disulfiram-Cu^{2+}, as measured by CCK8.

(**d**) Tumor weights of xenografts formed in BALB/c nude mice (n = 6) with KYSE30 cells.

(**e, f**) Subcutaneous tumors of nude mice with KYSE30 cells transfected with the indicated shRNAs (n = 6). Mice were treated with or without elesclomol. Tumor volume and weight were measured.

(**g**) Relative copper levels in xenograft tumors derived from different KYSE30 cells, with or without elesclomol treatment (n = 5).

Data are presented as mean ± SD; *P* value was calculated by two-way ANOVA (a-c and e) and Student's *t* test (d, f, and g).

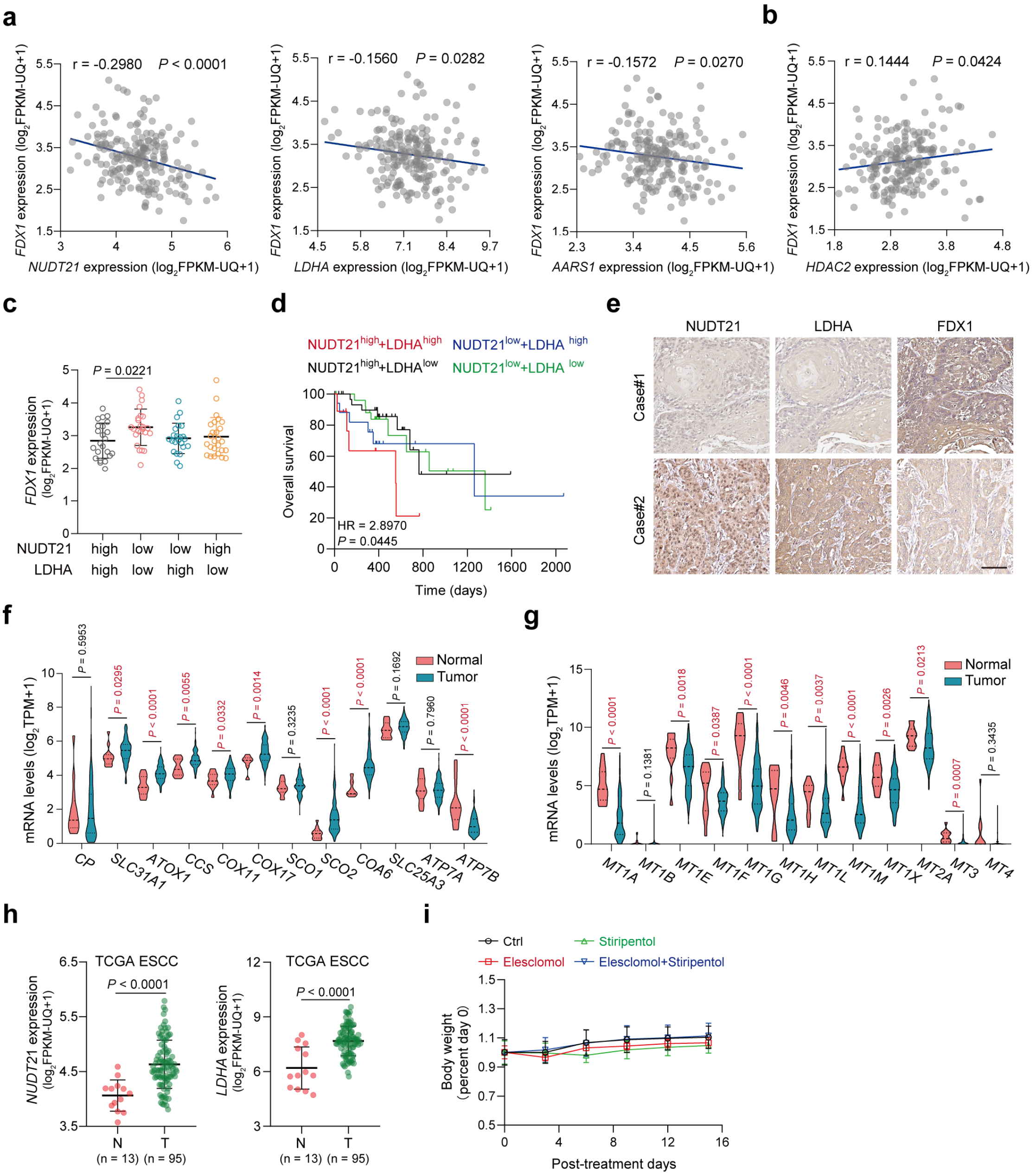

Fig. S10 The lactate–NUDT21–FDX1–cuproptosis axis as a potential therapeutic target.

(**a**) Scatter plots illustrating negative correlations between *FDX1* mRNA levels and those of *NUDT21*, *LDHA*, and *AARS1* in the esophageal cancer cohort.

(**b**) Scatter plots illustrating a positive correlation between *FDX1* and *HDAC2* in the esophageal cancer cohort.

(**c**) The mRNA expression levels of *FDX1* in the TCGA ESCC cohort stratified by *NUDT21* and *LDHA* expression levels.

(**d**) High expression levels of *NUDT21* and *LDHA* are correlated with the lowest overall

survival rate in the ESCC cohort.

(**e**) Representative IHC images of NUDT21, LDHA, and FDX1 staining in serial ESCC sections. Scale bars, 100 μm.

(**f, g**) The expression levels of genes involved in copper homeostasis (f) and copper storage (g) in tumors versus normal samples from the TCGA ESCC cohort. Genes with significant differences ($P < 0.05$) were highlighted in red.

(**h**) The mRNA expression levels of *NUDT21* and *LDHA* in the TCGA ESCC cohort.

(**i**) Mouse body weights were measured to assess the side effects of treatments.

Data are presented as mean ± SD; *P* value was calculated by Pearson correlation analysis (a, b), Student's *t* test (c, f, g, and h), and log-rank test (d).

Supplementary Table S1. The antibodies used in this study

Name	Manufacturer	Lot. No.	Source	Application	RRID
NUDT21	Proteintech	10322-1-AP	Rabbit	WB/IHC/IP/RIP	AB_2251496
FDX1	Proteintech	12592-1-AP	Rabbit	WB/IHC	AB_11182486
LDHA	Proteintech	19987-1-AP	Rabbit	WB/IHC	AB_10646429
Klac	PTM Bio	1401RM	Rabbit	WB	AB_2942013
Kac	ABclonal	A2391	Rabbit	WB	AB_2764337
ACTB	Proteintech	66009-1-IG	Mouse	WB	AB_2687938
FAM98B	Proteintech	22251-1-AP	Rabbit	WB	AB_2879049
Ub	CST	3936S	Mouse	WB	AB_331292
BTF3L4	Proteintech	F1804	Rabbit	WB	AB_2878265
Flag	CST	14793S	Rabbit	WB/RIP	AB_2572291
Flag	sigma	F1804	Mouse	PLA	AB_262044
myc	Proteintech	16286-1-AP	Rabbit	WB	AB_11182162
PAWR	Proteintech	20688-1-AP	Rabbit	WB	AB_10733473
HA	CST	3724S	Rabbit	WB	AB_1549585
CPSF6	Proteintech	15489-1-AP	Rabbit	WB/PLA	AB_10694140
AARS1	Proteintech	17394-1-AP	Rabbit	WB	AB_2219748
α-tubulin	Proteintech	66031-1-lg	Mouse	WB	AB_11042766
HDAC2	Abcam	ab219053	Rabbit	WB	
GAPDH	Proteintech	60004-1-IG	Rabbit	WB	AB_2107436
GFP	Proteintech	66002-1-Ig	Mouse	WB	AB_11182611
TRIP12	Proteintech	25303-1-AP	Rabbit	WB	AB_2880020
HDAC1	Abcam	ab150399	Rabbit	WB	
HDAC3	CST	D2O1K	Rabbit	WB	AB_2800047
SIRT1	Proteintech	13161-1-AP	Rabbit	WB	AB_10646436
SIRT2	Proteintech	19655-1-AP	Rabbit	WB	AB_2878592
SIRT3	Proteintech	10099-1-AP	Rabbit	WB	AB_2239240
LC3	Selleck	F0144	Rabbit	WB	

Supplementary Table S2. The primer sequences used for RT-PCR

Gene	Forward primer 5'-3'	Reverse primer 5'-3'
FDX1	CCCTGGCTTGTTCAACCTGT	CCCAACCGTGATCTGTCTGTTA
FDX1 dPAS	AGGCAGAGATCTAACCTGGC	AAGACACAGGTTCTAGCACAG
PAWR	TGCCGCAGAGTGCTTAGATG	CCTGTAGCAGATAGGAACTGCC
PAWR dPAS	GCAGTGGCTCATGCCTGTAA	AAGCACCTAACACAGCCTGG
FAM98B	TGGAGGCGCTGGGGTATAAA	GAGAGCCTAACCAAATACAGAGC
FAM98B dPAS	ACCCTTGAGCATGTTGTGTCT	ACAGGTGAGGCAAGGGTCTA
BTF3L4	AACAAGCCTTAGGAAGTTAGCTG	GCTTTACTGTCCAAGACTTGCC
BTF3L4 dPAS	TTCCTCTACCCTCCACCTGT	GTGGTTCCCCCTAACTCTGC
ACTB	CCAACCGCGAGAAGATGA	TCCATCACGATGCCAGTG

Supplementary Table S3. The oligonucleotide sequence of siRNAs and shRNAs.

genes	Sequence (5'-3')
siFDX1#1	ATGGACAATATGACTGTTCGA
siFDX1#2	GGACAATATGACTGTTCGA
siHDAC2#1	CCGTAATGTTGCTCGATGT
siHDAC2#2	GACCCATAACTTGCTGTTA
siCPSF6	GTTGTAACTCCATGCAATAAA
siAARS1#1	GCAGTGAGATCCACTACGA
siAARS1#2	GTTTGGCATTCCCATTGAA
shNUDT21#1	GAACCTCCTCAGTATCCATAT
shNUDT21#2	TGTACCCTCTTACCAATTATA
shFDX1	ATGGACAATATGACTGTTCGA
